# Multifunctional phototheranostic nanomedicine for cancer imaging and treatment

**DOI:** 10.1016/j.mtbio.2019.100035

**Published:** 2019-11-06

**Authors:** D. Gao, X. Guo, X. Zhang, S. Chen, Y. Wang, T. Chen, G. Huang, Y. Gao, Z. Tian, Z. Yang

**Affiliations:** a The Key Laboratory of Biomedical Information Engineering of Ministry of Education, School of Life Science and Technology, Xi'an Jiaotong University, Xi'an, 710049, China; b John A. Paulson School of Engineering and Applied Sciences, Harvard University, Cambridge, MA, 02138, USA; c Henan Provincial People's Hospital, Zhengzhou University People's Hospital, Number 7 Weiwu Road, Zhengzhou, 450003, China; d State Key Laboratory of Non-food Biomass and Enzyme Technology, Guangxi Academy of Sciences, Nanning, 530007, China

**Keywords:** Theranostics, Nanoparticles, Photodynamic therapy, Photothermal therapy, Cancer treatment

## Abstract

Cancer, as one of the most life-threatening diseases, shows a high fatality rate around the world. When improving the therapeutic efficacy of conventional cancer treatments, researchers also conduct extensive studies into alternative therapeutic approaches, which are safe, valid, and economical. Phototherapies, including photodynamic therapy (PDT) and photothermal therapy (PTT), are tumor-ablative and function-reserving oncologic interventions, showing strong potential in clinical cancer treatment. During phototherapies, the non-toxic phototherapeutic agents can be activated upon light irradiation to induce cell death without causing much damage to normal tissues. Besides, with the rapid development of nanotechnology in the past decades, phototheranostic nanomedicine also has attracted tremendous interests aiming to continuously refine their performance. Herein, we reviewed the recent progress of phototheranostic nanomedicine for improved cancer therapy. After a brief introduction of the therapeutic principles and related phototherapeutic agents for PDT and PTT, the existing works on developing of phototheranostic nanomedicine by mainly focusing on their categories and applications, particularly on phototherapy-synergized cancer immunotherapy, are comprehensively reviewed. More importantly, a brief conclusion and future challenges of phototheranostic nanomedicine from our point of view are delivered in the last part of this article.

## 1 Introduction

Cancer, as one of the most life-threatening diseases, is predicted to rank as the primary reason for death and the biggest obstacle to extend life span over the next decades. In accordance with the status report provided by the International Agency for Research on Cancer, there were an estimated 18.1 million new cases of cancer and 9.6 million deaths from cancer worldwide in 2018 [1]. Moreover, it is expected that the number of new cases will continuously increase in the future 20 years [2,3]. Although some conventional cancer treatments, such as surgery, chemotherapy, and radiotherapy have been widely applied in the clinic, they still cannot completely eradicate tumors, whereas cause severe side-effects. Nowadays, when improving the therapeutic efficacy of currently existing treatments in the clinic, researchers also devote to developing alternative approaches for cancer imaging and therapy, which are safe, valid, and economical.

**Fig. 1:**
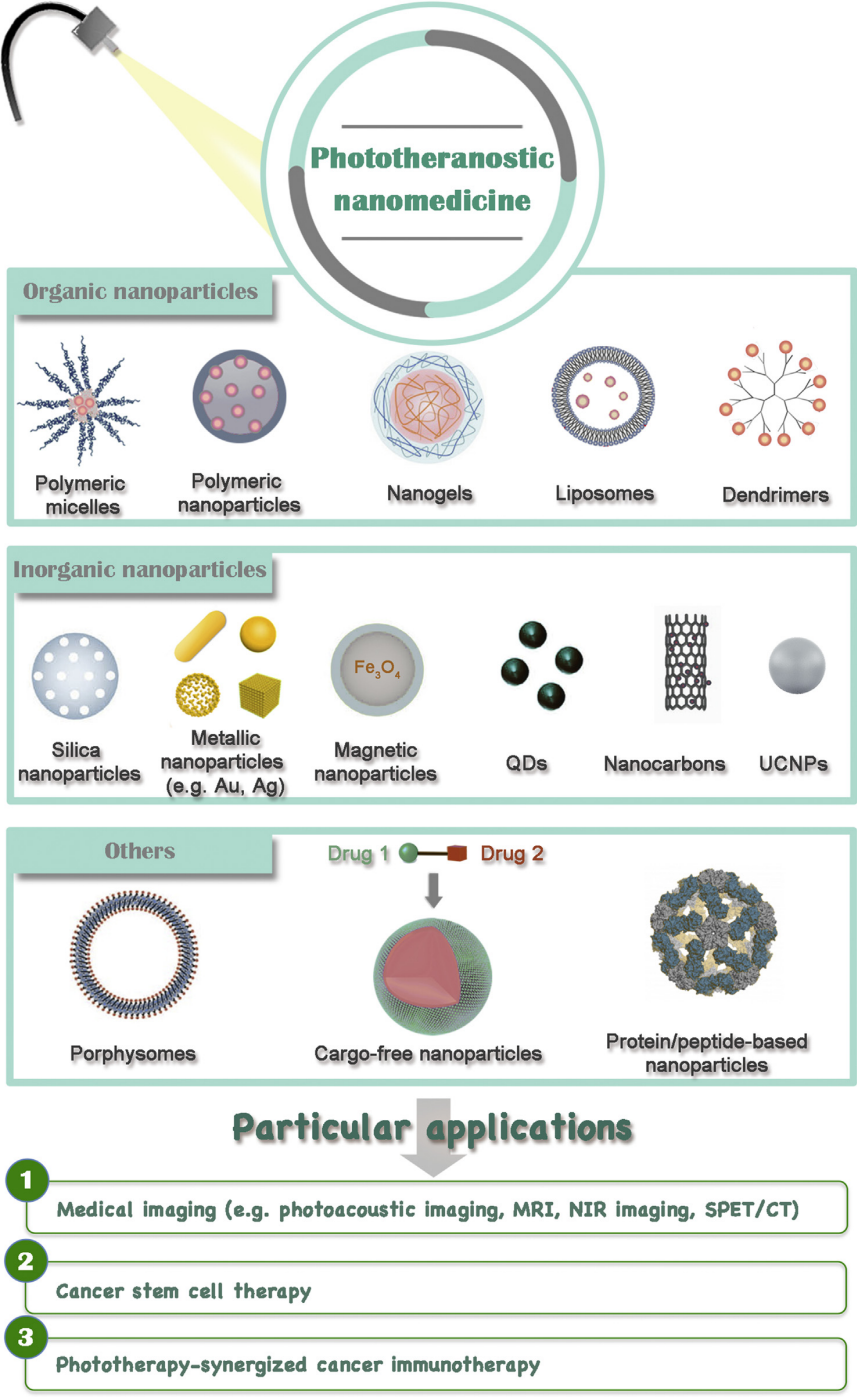
Overview of categories and particular applications of phototheranostic nanomedicine.

Phototherapies, including photodynamic therapy (PDT) and photothermal therapy (PTT), are tumor-ablative and function-reserving oncologic interventions, showing great potential in clinical cancer therapy. In the process of phototherapies, the non-toxic phototherapeutic agents can be activated upon light irradiation, resulting in selectively killing cancer cells without inducing severe side-effects. Through the meticulous design of phototherapeutic agents and well control of light illumination in the location of lesions (e.g. tumor tissues), the dual ‘selectivity’ in phototherapies could be achieved with a consequence of reducing the systemic toxicity involved in traditional chemotherapy and radiotherapy [4,5]. Moreover, with the rapid development of nanotechnology in the past decade, combining phototherapies and nanomedicine, termed phototheranostic nanomedicine, has attracted tremendously increasing interests aiming to continuously refine the phototherapeutic efficacy. To get a better understanding of phototheranostic nanomedicine, some therapeutic principles and related phototherapeutic agents of PDT and PTT are introduced at the beginning of this review. Subsequently, the latest research advancements on phototheranostic nanomedicine are comprehensively reviewed (Fig. 1). Especially, research progresses on phototherapy-synergized cancer immunotherapy are emphatically discussed in this section owing to the ever-rising interests in clinical immunotherapy recently. Finally, the challenges and future perspectives of phototheranostic nanomedicine for advanced cancer treatment are presented.

### 1.1 Photodynamic therapy

**Fig. 2:**
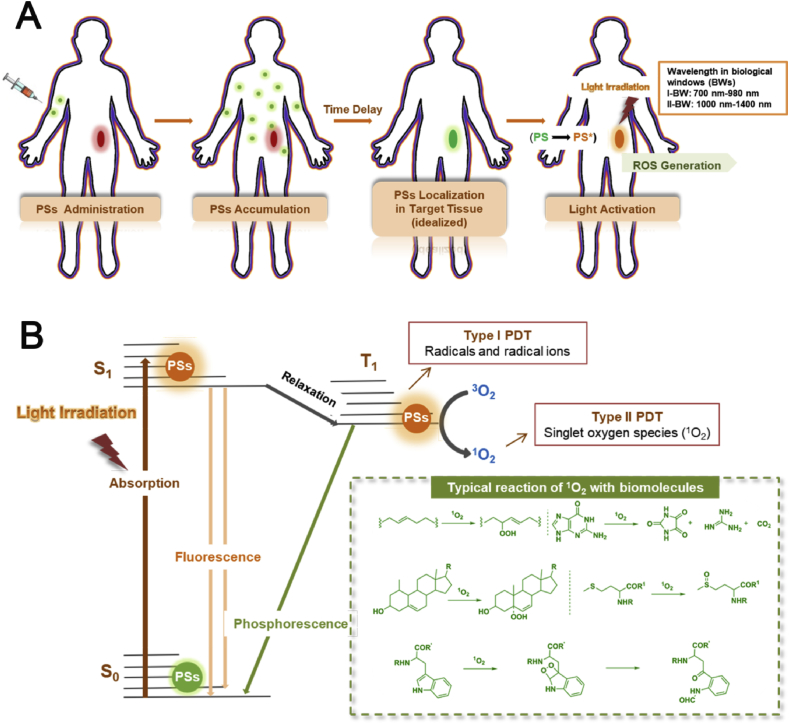
(A) Schematic illustration of the procedures during PDT. (B) Simplified Jablonski diagram showing a different photodynamic process and photochemical reaction (type I and type II) after activating the PSs by light irradiation, and typical reactions of ^1^O_2_ with selected biomolecules. PDT, photodynamic therapy; PSs, photosensitizers.

PDT is a photochemistry-based therapeutic modality combining the action of photosensitizers (PSs), light and oxygen molecules to produce reactive oxygen species (ROS), leading to the death of tumor cells [6,7]. In the process of PDT, the PSs are administrated either intravenously or topically to patients, followed by illuminating the disease location using optical fiber with an appropriate drug-light interval (Fig. 2A). To avoid interference from any endogenous chromophores inside the human body and enhance the penetration depth of light in tissues, the wavelength of light ranging in far-red/near-infrared (NIR) region is used for PDT. By choosing the light with longer wavelength, the application scope of cancer PDT could enlarge from superficial tumors to deeply located ones.

Light has been used in combination with a chemical agent for repigmentation of vitiligo in Egypt and India since about 3000 years ago [8]. Until 1993, Photofrin (PF), a hematoporphyrin (Hp) derivative, was firstly approved for PDT in Canada to treat bladder cancer in some specific cases [9]. Two years later, the Food and Drug Administration (FDA) approved this therapeutic approach in the United States for the mitigation of obstructive esophageal cancer [9]. Nowadays, there are several advancing clinical researches and trials for various types of cancers and age-related macular degeneration (AMD) by using the different principles of PDT [10].

#### 1.1.1 Principle of PDT

Although a large amount of PSs (Section 1.1.2) has been used for PDT, the general photophysical mechanisms and biological mechanisms are similar, which are summarized in the following subsections.

##### 1.1.1.1 Photophysical mechanisms

Upon illumination, the PSs are promoted to their excited singlet state (S_1_, higher energy orbital) from their ground state (S_0_, lower-energy orbital) by absorbing a photon and exciting an electron. As the S_1_ state is highly unstable and short-lived state, the excited PSs prefer to return to S_0_ state immediately, dissipating their energy by fluorescence emission or non-radiative vibrational relaxation, which could be used for diagnosis. Alternatively, the PSs can be populated to an unstable excited triplet state (T_1_) through the intersystem crossing. To dissipate the energy in the T_1_ state, the PSs can emit phosphorescence or react with different molecules to generate reactive species via two types of photochemical reactions (type I and type II). In the type I reaction, the excited PSs can directly react with other biomolecules to form radicals or radical ions via various redox reactions, that subsequently react with oxygen to form other ROS, such as superoxide anion radicals (^•^O_2_^-^) and hydrogen peroxide (H_2_O_2_), leading to the cell death directly. In the type II reaction, PSs in T_1_ could react with ground state oxygen (^3^O_2_) to form high cytotoxic singlet oxygen (^1^O_2_) by energy transfer (Fig. 2B) [6]. In the presence of plentiful ^1^O_2_, some typical reactions (inset in Fig. 2B) of ^1^O_2_ with biomolecules could be induced, further causing cell death to realize the PDT effect. Owing to the spin-allowed nature of this dissipation, a majority of PSs are proved to experience type II reactions during the PDT process. However, it has been reported that type I and type II reactions can be modulated through tuning the external factors, such as oxygen level and electron density of surroundings [11].

##### 1.1.1.2 Biological mechanisms

In biological systems, the ROS generated during the PDT process can induce various biological responses, depending on the localization of PSs in the tumor tissues. The short lifetime of the ROS limits their diffusion distance (20–300 ​nm) [6]. Based on the current research, PDT can induce damage on cancer cells and tumor vasculature and activate the immune response of the host: (1) Damage on cancer cells: when the PSs localize in the cancer cells, PDT can induce two cell death pathways, including cell necrosis and cell apoptosis. If there is an ROS accumulation in cancer cells or severe rupture on the cell membrane during PDT, the cells will undergo necrosis uncontrollably and physically [12]. On the other hand, PDT can induce cell apoptosis at the cellular level, which is a programmed cell death pathway. In this case, ROS generally damages cell organelles, promoting cell apoptosis via activating several signaling pathways [13,14]. (2) Damage on tumor vasculature: the fast growth of tumors relies on the provision of nutrients through tumor vasculatures. Different targeted PSs have been developed to break vasculatures and shut down the nutrient supply after PDT [15]. (3) Inducing immune response: PDT was considered as a topical treatment initially until extensive studies ascertained that photosensitization could also activate the innate and adaptive immunity and generate antitumor immunity. It appears that the PDT process can cause expression of endogenous danger signals which are referred to as damage-associated molecular patterns (DAMPs) [16]. After interacting with pattern recognition receptors, for example, toll-like receptors expressed on innate immune cells, the DAMPs change to be immunostimulatory, which are capable of activating antigen-presenting cells, promoting dendritic cells (DCs) maturation [17,18] and further generating CD8^+^ effector and memory T cells. In addition, PDT can also diminish tumor-derived immunosuppression. Immunosuppressive cells, such as T_regulatory_ cells (T_reg_) and myeloid-derived suppressor cells, could be destructed during PDT, further potentiating the antitumor immunity [19]. Moreover, the non-specific immune response could also be induced by cell necrosis; hence promoting the accumulation of white blood cells to defend the tumor growth.

#### 1.1.2 PSs for PDT

PSs play an essential role in PDT, significantly influencing the treatment effect. An ideal PS should fulfill several requirements. First, the PSs should possess strong absorption in far-red/NIR region. In the human body, there is a wide range of biomolecules capable of absorbing light at a shorter wavelength (<650 ​nm), such as hemoglobin (Hb), significantly limiting the penetration of light in tissues. To avoid the interference from these biomolecules, the NIR light is preferred to irradiate the PSs to enhance tissue penetration to a few centimeters [20]. Second, the tumor-targeting effect of PSs should be taken into consideration. In accordance with the previous studies, non-specific localization and uptake of PSs could lead to the undesired skin toxicity [21]. Moreover, the PSs should be safe, biodegradable, and biocompatible. Finally, the high ROS production efficiency is another prerequisite for PSs [22].

**Fig. 3:**
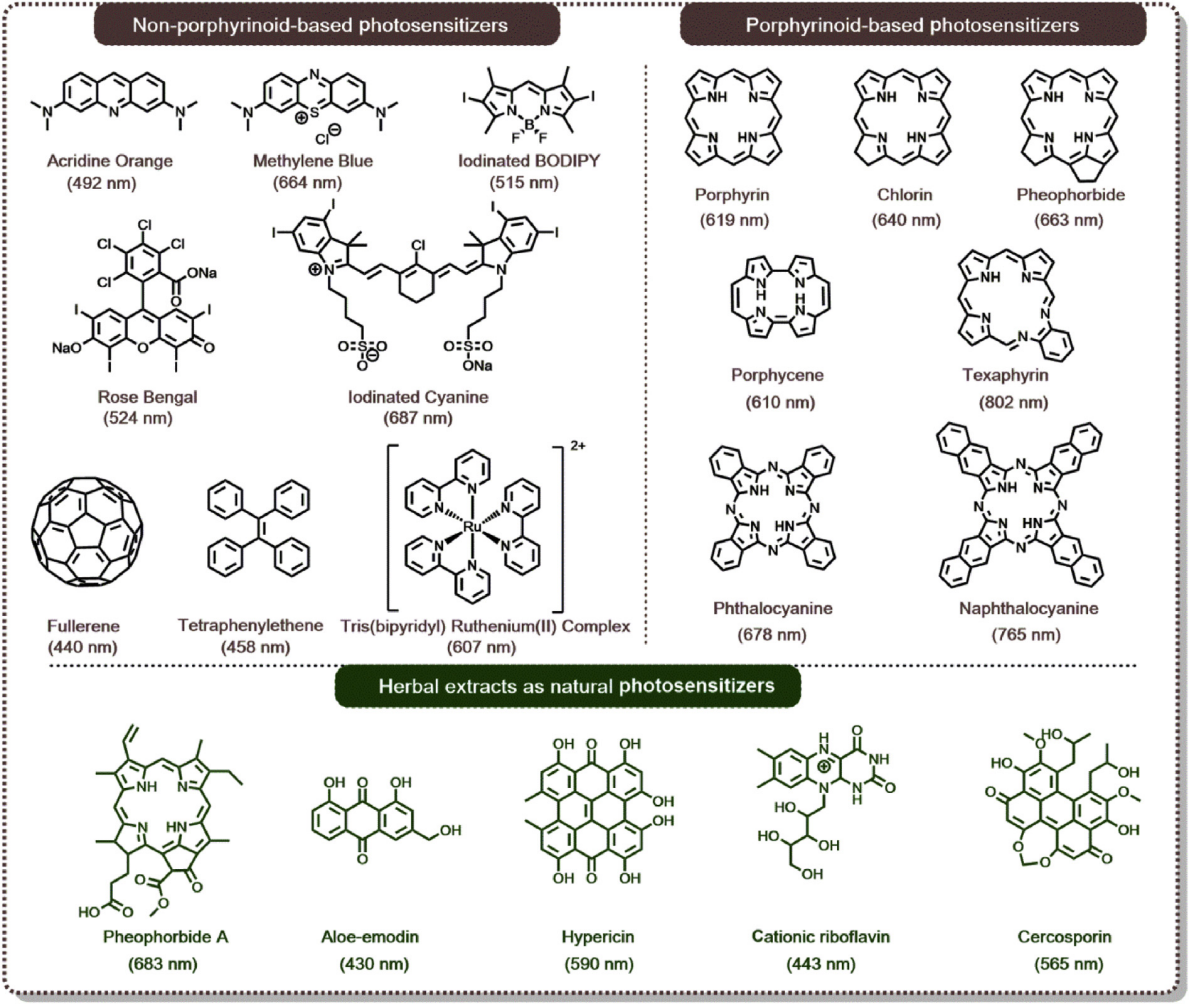
Representatives of non-porphyrinoid, porphyrinoid-based PSs, and natural PSs extracted from herbals. The absorption peak of PSs corresponding to the excitation wavelength of light for PDT was highlighted with parentheses below each PS. PDT, photodynamic therapy; PSs, photosensitizers.

Generally, the PSs can be divided into two classes, which are non-porphyrinoid-based PSs and porphyrinoid-based PSs (Fig. 3). Some representative non-porphyrinoid-based PSs, such as acridine orange [23], rose bengal (RB) [24,25], and methylene blue (MB) [26] were used for PDT during the dipyrromethene boron difluoride (BODIPY) derivates [27], ruthenium (II) complexes [28], tetraphenylethene derivates, [29] and fullerene [30] have received more attention. In addition, porphyrinoid-based PSs have also been widely studied, including porphyrins [31], chlorins, pheophorbides, texaphyrins, porphycenes, phthalocyanines (Pc), naphthalocyanines (Nc) and so on [32]. These PSs could be prepared and modified readily. Moreover, they exhibited negligible dark cytotoxicity because of their similar structures to Hb, a porphyrin moiety in the human body [33]. In 1913, Hp was firstly administrated to the patients. Since then, other porphyrinoid-based PSs or their metabolic precursors were explored for the clinical application (Table 1).

**Table 1:** Summary of PSs approved for clinical application [34].

PS	Excitation wavelength	Approved	Indication
Porfimer sodium/PF®	630 ​nm	Worldwide, withdrawn in EU for commercial reasons	Severe dysplasia in Barrett's esophagus. Obstructive esophageal or lung cancer
5-ALA/Ameluz®/Levulan®	635 ​nm	Worldwide	Mild to moderate actinic keratosis
Metvix®/Metvixia®	570–670 ​nm	Worldwide	Non-hyperkeratotic actinic keratosis and basal cell carcinoma
Temoporfin/mTHPC/Foscan®	652 ​nm	Europe	Advanced head and neck cancer
Talaporfin/NPe6/Laserphyrin®	664 ​nm	Japan	Early centrally located lung cancer
Verteporfin/Visudyne®	690 ​nm	Worldwide	AMD
Redaporfin®/LUZ11	749 ​nm	Orphan status in EU	Biliary tract cancer
Synthetic hypericin/SGX301	570–650 ​nm	Orphan status in EU	Cutaneous T-cell lymphoma

PS, photosensitizer; PF, photofrin; AMD, age-related macular degeneration; NP, nanoparticle; EU, European Union; mTHPC, meta-tetra (hydroxyphenyl) chlorine.

Recently, some photosensitizing compounds extracted from herbal medicine plants in combination with light activation were proved to exert an anticancer effect, making them alternative PSs for PDT. These compounds include pheophorbide A, tolyporphin, chlorophyllin, curcumin, anthraquinones, cationic riboflavin, hypericin, hypocrellin, cercosporin, aloe emodin and so on (partially displayed in Fig. 3) [[35], [36], [37]]. Compared with synthetic PSs, natural ones are ubiquitous and more readily accessible. Furthermore, natural PSs usually induce fewer side-effects than other routinely used PSs. In accordance with the recent studies, natural PSs may be considered potential candidates for PDT, and it is also believed that researchers may discover more photoactive plants in nature to extend the source of this special category of PSs.

To finish on a lighter note, most widely studied PSs are operated in the visible or NIR I region (700–900 ​nm) at present. To optimize the spatial resolution, improve the signal-to-noise ratio, and enhance the tissue penetration depth for biological imaging, it is quite necessary to develop another class of PSs, which can be excited in the NIR II region (1000–1700 ​nm) [38,39]. Li et al. [40] ​reported to use the tungsten carbide NPs (W_2_C NPs) for both type I and type II PDT upon irradiated by a 1064-nm laser. Accordingly, because the energy gap is relatively small for these W_2_C NPs, it is easy to create electron-hole pairs via interband transitions, finally oxidizing substrates to generate OH⋅ and ^1^O_2_ for PDT. Besides, W_2_C NPs could also convert the light energy to heat energy under 1064-nm laser illumination for PTT. Ultrasmall Cu_2-x_Se NPs showed strong absorbance in the NIR II region. Zhang et al. [41] ​developed the novel drug-loaded ultrasmall Cu_2-x_Se theranostic NPs for photoacoustic (PA) imaging–guided chemo-PDT against orthotopic malignant glioblastoma, and the tumor growth was completely inhibited based on the results *in vivo*. However, even several NIR II PSs have been developed, most of them are inorganic metallic NPs. Therefore, there is still a long way to go before applying these PSs in the clinic and the organic-based ones are ready to come out at their call.

### 1.2 Photothermal therapy

#### 1.2.1 General concept in PTT

It is well recognized that temperature is crucial to adjust the activity and viability of biological systems in cells and tissues. Any uncontrollably enhanced temperature above the normal body temperature (37 ​°C) can cause fever and induce severe damage to our body, such as fatal organ failure. On the other hand, a well-controlled enhancement of temperature can also be beneficial to patients, such as ones with cancer, which was first reported in the 19th century [42]. In recent decades, the interests in thermal treatment of cancers have been reactivated because of the advances of techniques in controlling heating. In addition, researchers have a better understanding of thermal therapy, such as the mechanism for temperature-induced cell killing and modification [43]. Accordingly, thermal therapy can be classified into irreversible injury treatments (48–60 ​°C), hyperthermia treatments (41–48 ​°C), and diathermia treatments (37–41 ​°C), depending on the extent of the temperature increment [44]. Moreover, there have already been several ways to accelerate the enhancement of tissue temperatures, such as light irradiation, the action of an alternating magnetic field (magnetic hyperthermia) [45], and microwave radiation (microwave hyperthermia) [46].

PTT, which utilizes light to induce hyperthermia/thermal ablation, has been recognized as an uncertain technique at first because of the strong extinction coefficients of human tissues within the visible range of the optical spectrum. However, the PTT would also induce damage to the normal tissues nearby the heat location. To solve this problem, laser irradiation with optical fiber, termed as laser hyperthermia, has been developed to enhance the targeting effect through direct delivery of the light to the tumor tissues [47]. Moreover, PTT agents with a high absorption coefficient in the NIR regions have been applied to further improve the performance of PTT in cancer treatment [48]. After being administrated into the tumor sites, the PTT agents could efficiently perform the photothermal conversion to induce the temperature increase at the tumor sites. And the photothermal conversion efficiency of PTT was closely relevant to the NIR absorption wavelength, the absorption coefficient of PTT agents and the power of the excitation lights [49].

#### 1.2.2 PTT agents for PTT

Excitingly, in August 2019, Rastinehad et al. [50] reported the initial results of a clinical trial in which gold-silica nanoshells (GSNs) were used as PTT agents associated with magnetic resonance-ultrasound fusion imaging to ablate low-intermediate-grade tumors within the prostate. As per their report, GSN-mediated thermal therapy was successful in 94% (15/16) of patients, showing no significant difference in the International Prostate Symptom Score or Sexual Health Inventory for Men. It is the first time for researchers to present the results of clinical trials on treating cancer by PTT in an authoritative journal. Owing to the unceasing research on PTT agents, many research groups can prepare various PTT agents with promising performance. These PTT agents have fulfilled the requirements, such as strong absorption within biological windows (BWs, BW I: 700 ​nm–980 nm/BW II: 1000 ​nm–1400 nm) [51], low toxicity, easy functionalization, and good solubility in biocompatible liquids. Nowadays, some NPs and organic molecules, satisfying the requirements aforementioned, have already been applied in PTT. This section will briefly review the characters of these PTT agents and their applications in cancer therapy.

##### 1.2.2.1 Metallic NPs

Noble metal NPs have been widely investigated because of their promising characters such as large optical field enhancements and high photothermal conversion efficiency. The valence electrons of the metal NPs could go through a lattice-related oscillation upon light irradiation [52]. Based on Mie theory, this oscillation, also known as localized surface plasmon resonance (LSPR), is defined as the resonance at specific frequency relevant to the magnetic field of light [53]. The LSPR wavelength of gold and silver NPs is in the range of visible light to NIR light. Moreover, there are several important parameters affecting the LSPR properties of the NPs, such as size, shape, interparticle distance, the kinds of metal, and the local dielectric constant. Different plasmonic NPs with tunable LSPR have been prepared and their strong enhancement in the light absorption leads to the obvious photothermal effect under light irradiation, making them promising PTT agents for cancer treatment. As the most significant type of metallic nanoparticles, gold nanoparticles (GNPs), such as gold nanorods (GNRs), gold nanocages (GNCs), and gold nanoshells (GNSs), were prepared and evaluated as PTT agents for cancer treatment over the last decade [[54], [55], [56]].

###### 1.2.2.1.1 Gold nanorods (GNRs)

**Fig. 4:**
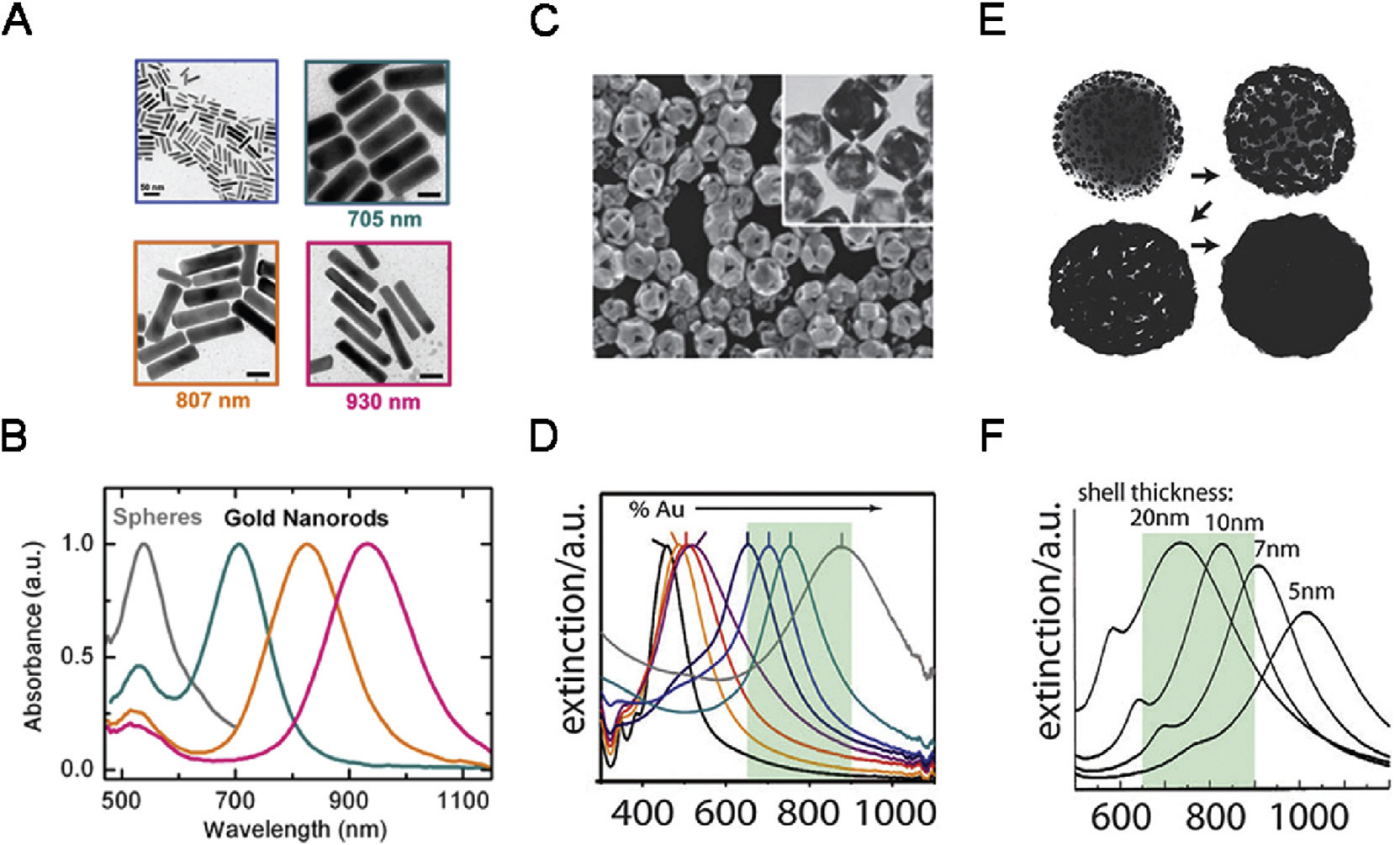
TEM images and absorption spectra of typical GNRs (A and B) [71], GNCs (C and D), and GNSs (E and F) [72]. GNRs, gold nanorods; GNCs, gold nanocages; GNS, gold nanoshells.

The GNRs are extensively studied for PTT because of their excellent absorption in the NIR region [57]. As shown in Fig. 4A and B, GNRs are elongated in shape and exhibit two different surface plasmon bands, the transverse SPR and longitudinal SPR [58]. GNRs could be prepared by a seed-mediated method by using cetyltrimethylammonium bromide (CTAB) as the cationic surfactant. Through adjusting the feeding ratio of the agents during the preparation of GNRs, the aspect ratio of GNRs could be controlled. However, there is still a constraint on the clinical use of GNRs during PTT, which is the cytotoxicity induced by CTAB, forming a charged bilayer facing outward. To reduce the cytotoxicity induced by CTAB, the surface of GNRs could be further modified by other less toxic molecules or coated with some other materials, such as polyethylene glycol (PEG) [59,60], thiolated polyamidoamine (PAMAM) dendrimers [61], chitosan [62], mesoporous silica nanoparticles (MSNs) [63], MoS_2_ [64] and so on.

###### 1.2.2.1.2 Gold nanocages (GNCs)

The GNCs are usually prepared by a galvanic replacement reaction first ​mentioned in 2002 [65]. In this method, silver nanocubes are used as the templates for the nucleation of reduced gold atoms and further growth. It costs three silver atoms to reduce one gold atom leading to the generation of holes in the walls of the cubes. The amount of gold salt fed during synthesis significantly influences the thickness of the wall and the size of the holes on the GNCs (Fig. 4C and D). The therapeutic applications of GNCs have been investigated. In 2007, Chen et al. [66] conjugated anti-human epidermal growth factor receptor-2 (HER2) antibodies to the GNCs for the PTT on HER2-overexpressed breast cancer cells and the cell viability decreased significantly upon laser irradiation.

###### 1.2.2.1.3 Gold nanoshells (GNSs)

GNSs are comprised a thin gold shell and a hollow cavity, in which their plasmonic absorption could be tuned by adjusting the ratio of the shell thickness to the core radius (Fig. 4E and F) [67]. The GNSs were first ​discovered in 1989 [68] and synthesized by Oldenburg et al. [69] in 1998. During the synthesis, negatively charged GNPs were first ​electrostatically adsorbed onto the surface of silica NPs with primary amine groups. Then, the obtained NPs were utilized as nucleation spots for the further chemical reduction of gold, finally preparing a conformal shell coating on the silica core. To improve their biocompatibility and dispersity in the aqueous solution, the surface of the GNSs could be modified by PEG [70].

##### 1.2.2.2 Semiconductor quantum dots

Quantum dots (QDs) are kinds of semiconductor nanocrystals being widely used for fluorescence imaging. In the beginning, various semiconductors with direct band gaps have been identified as the semiconductors QDs, including CdSe, CdTe, CuSe, CuS, InP and so on, showing strong fluorescence emission upon excited by the visible light or NIR light [72]. Hence, these QDs have been applied for high-contrast *in vitro* and *in vivo* imaging [73]. Accordingly, the host semiconductor material and their size, capable of inducing the quantum confinement effects, determine the optical characters of QDs. Owing to the unique properties of QDs, they could not only be used as fluorescence probe through tailoring their emission wavelength but also as multifunctional fluorescent probes capable of intracellular thermal sensing during hyperthermia treatments [74]. To further enhance the extinction cross-sections, which is a limiting factor for QDs, researchers are focusing on preparing more novel QDs. Lakshmanan et al. [75] synthesized Au/CuS nanocomposites that were composed of a CuS core and an Au shell. The plasmonic-induced local field enhancement elevated the extinction coefficient of CuS-QDs in the Au/CuS by twice in such cases.

##### 1.2.2.3 Rare-earth ions–doped nanocrystals

Rare-earth ions doped nanocrystals are promising fluorescent nanoprobes with wide applications from fluorescence bioimaging to thermal sensing [76]. Because the rare-earth ions have the unique electronic configuration, they usually display a rich energy level diagram. Through incorporating these ions into dielectric materials, there are some extra energy levels that appeared within the band gaps, resulting in the occurrence of narrow absorption bands. Normally, upon proper light irradiation, electrons in the ground state could be excited to their excited states, followed by the relaxation back to the ground state immediately via radiative or non-radiative processes (heat generation). Besides, the relaxation dynamics in rare-earth ions–doped nanocrystals have been proved to be more complex with the enhancement of the rare-earth ion content. Owing to the reduced distances between neighboring rare-earth ions in this situation, ion-ion interactions could be activated. Therefore, the heat could be generated based on the dual mechanisms including cross-relaxation and energy migration [77]. For example, NPs incorporating neodymium or ytterbium/erbium ions showed excellent light-to-heat conversion efficiency, considered as promising PTT agents for further applications [78].

##### 1.2.2.4 Carbon-based NPs

Among various carbon-based nanostructures, graphite-related ones, such as single-walled carbon nanotubes (SWCNTs), multiwalled carbon nanotubes and graphene nanoparticles (GphNPs), have shown remarkable photothermal conversion efficiency, acting as efficient PTT agents for biomedical application. In 1991, Iijima et al. [79] ​discovered a kind of cylinder ​composed of sheets of Gph and named them carbon nanotubes (CNTs). As for the morphology of CNTs, they are in a tube shape with several nanometers in diameter. Besides, their lengths could change from several nanometers to microns. Accordingly, the de-excitation process of CNTs mainly includes luminescence and non-radiative relaxation. Usually, the fluorescence of CNTs is significantly quenched becasue of the mutual effect between different carbon layers or carbon layers with additional components, resulting in low fluorescence quantum yield. As a consequence, most of the energy absorbed from light irradiation could be converted into heat [80]. In addition, the mechanism of light-triggered collective movement of free carriers could also induce the hyperthermia of CNTs. The way of heat generation is similar to the GNPs, which is the relaxation of surface electrons. When compared with GNPs, carbon-based NPs show significant extinction among the whole biological spectral range. Therefore, carbon-based NPs display the superiorities of tunable PTT in a wide spectral range and that is the main reason for these nanostructures to be used for PTT [81,82].

##### 1.2.2.5 Organic molecular–based photothermal agents

In recent years, organic molecular–based PTT agents have attracted much attention when performing PTT. Compared with the aforementioned inorganic counterparts, organic-based PTT agents could be designed to achieve the safety, cancer-targeting effect, and multifunctionality through dedicated synthesis [83]. Upon light excitation, the electrons of organic PTT agents can be excited to the excited singlet state and then undergo an internal conversion to the lowest excited singlet state (S_1_). Meanwhile, the photothermal effects could be induced by the non-radiative relaxation processes because of the collisions between the excited singlet species and its surroundings. As for these PTT agents, they should fulfill the following requirements for further clinical application, including strong absorption in the NIR region, minimized fluorescence quantum yield and ROS generation efficiency, non-toxic in dark, biodegradable and biocompatible, and ease of chemical synthesis [84]. So far, various organic PTT agents have been developed and used for cancer therapy, including cyanines, diketopyrrolopyrroles, porphyrins, and some polymers.

###### 1.2.2.5.1 Cyanine-based agents

**Fig. 5:**
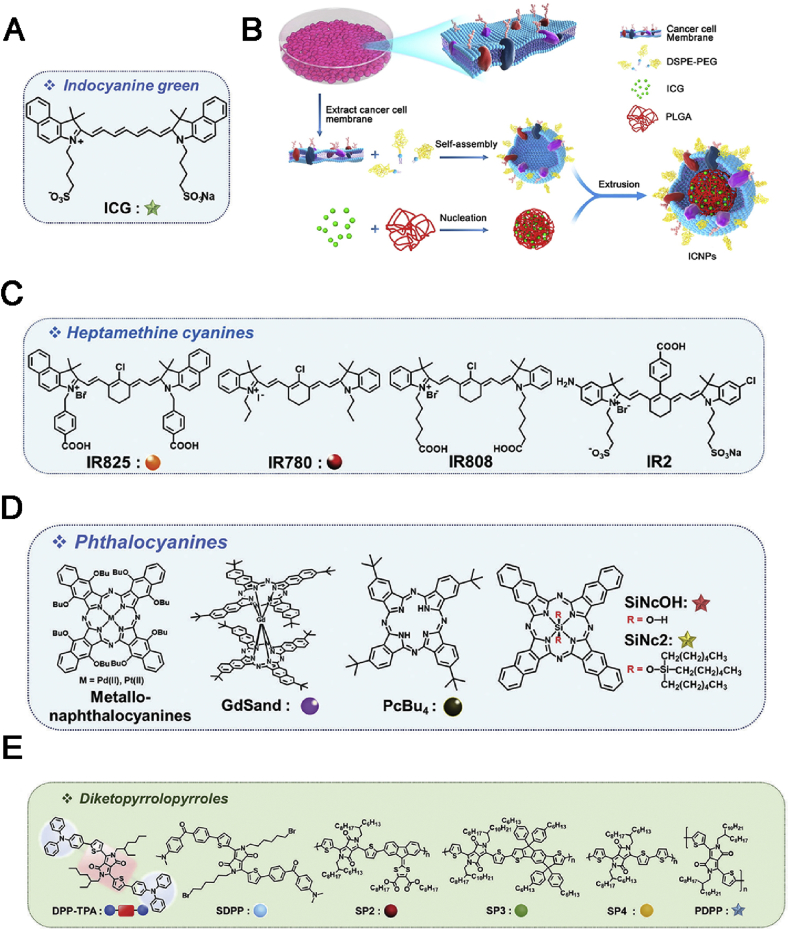
(A) Chemical structure of ICG [84]. (B) Illustration of ICG/PLGA cancer cell membrane coated biomimetic agent [87]. (C) Chemical structures of various heptamethine cyanines, including IR825, IR780, IR808, and IR2 [84]. (D) Chemical structures of Pcs, including metallo-Ncs, GdSand, PcBu4, SiNcOH, and SiNc2 [84]. (E) Molecular structure of diketopyrrolopyrroles: DPP–TPA, SDPP, SP2, SP3, SP4, and PDPP [84]. ICG, indocyanine green; PLGA, poly (lactic acid-co-glycolic acid); DPP, diketopyrrolopyrrole; GdSand, gadolinium-bisnapthalocyanine sandwich complex.

Among various cyanine-based NIR PTT agents, indocyanine green (ICG) (Fig. 5A) is the quintessential one since that it was approved by the FDA as an optical imaging agent. ICG can also efficiently generate ROS and convert light to heat. However, the clinical application of ICG was still limited because of its poor *in vivo* stability and non-specific biodistribution [84]. To overcome these issues, ICG was encapsulated into NPs to improve its performance [85,86]. Chen et al. [87] constructed a biocompatible PTT agent as shown in Fig. 5B. In their work, ICG was loaded into the poly (lactic acid-co-glycolic acid) (PLGA) NPs and further modified by a homologous cancer cell membrane. It was found that prolonged blood circulation and evaded immune response were observed owing to the presence of self-antigens on the surface of NPs. Moreover, these NPs can also be applied in cancer diagnosis becayuse of their functions of real-time fluorescence, PA imaging, and efficient PTT.

Heptamethine cyanines, ICG derivatives, have also been extensively studied for PTT. There are several heptamethine cyanines, such as IR825, IR780, IR808, and IR2 (Fig. 5C). To improve their aqueous solubility and photostability, various nanoformulations embedding these dyes have been developed for cancer PTT [88].

Besides, the aforementioned Pcs exhibit excellent optical stability, thermal stability, and preferable absorption coefficient in the NIR region [89]. Apart from generating ROS, it has been reported that phthalocyanines (Pcs) can also act as PTT agents. Owing to the special planar nature of Pcs along with their strong hydrophobicity, Pcs tend to aggregate orderly in aqueous solution, leading to the reduction of fluorescence and ROS generation. They further undergo an activation of non-radiative, heat-producing de-excitation pathways for PTT (Fig. 5D) [90,91].

###### 1.2.2.5.2 Diketopyrrolopyrrole-based agents

Owing ​to their superior properties, such as strong absorption in the NIR region and outstanding photostability, diketopyrrolopyrroles have been used for PTT, recently (Fig. 5E). They can convert photo-to-heat conversion when the fluorescence was completely quenched because of self-aggregation, especially for the donor-acceptor-donor systems. Based on their characteristics, several diketopyrrolopyrrole-based agents were developed either in the form of small molecules or encapsulated into nanocarriers [92, 93,94].

###### 1.2.2.5.3 Porphyrin-based agents

On the absorption spectra of typical porphyrins, it usually exhibits a high hypsochromic band (Soret band) and four low bathochromic bands (Q-band). With the purpose to improve the physical properties of porphyrins for PTT, it is necessary to shorten the excited state lifetime through self-assembly of porphyrin molecules into nanoformulations or introduction of certain metal cations into the porphyrin core [95,96]. In 2011, Lovell et al. [97] developed a fully biodegradable liposome-like NP, termed as porphysome, by self-assembly of the modified phospholipids, in which one of the phospholipid fatty acid chains was replaced by a porphyrin derivative (pyropheophorbide). This novel nanoplatform is specifically described in Section 2.6.1.

Overall, organic molecular–based PTT agents with low fluorescence quantum yield and singlet oxygen production efficiency can achieve the efficient thermal events upon NIR irradiation. Despite a large number of related literature that has been published in recent years, overall it seems the research in the field of organic molecular–based PTT agents is still in its infancy and there are still extensive challenges that need to be overcome to translate present developments into clinical trials. Especially, the optimal delivery of PTT agents to tumor tissues and sensitive subcellular compartments is essential to realize the successful PTT.

##### 1.2.2.6 Melanin-like materials–based PTT agents

Melanins are well recognized natural biopolymers that are widely distributed in many organisms including human skin. They were found to present a wide spectrum of functions in the biosystem, including antioxidation, photoprotection, thermoregulation, and some intervention in nervous systems [98]. Besides, owing to their inscape and various biological functions, melanins have been considered as a new class of biomaterials for biomedical applications. Melanins possess excellent photothermal conversion ability and can be used as PTT agents for cancer treatment. For example, Jiang et al. [99] ​prepared the red blood cell (RBC) membrane-camouflaged melanin NPs as a platform for effective *in vivo* antitumor PTT and obtained as high as 40% photothermal conversion efficiency.

However, most natural melanins are extracted from the pigment from their biological environment, and there is no ideal and standardized procedure to obtain melanins in nature without influencing their intrinsic physicochemical properties [98]. To solve this problem, poly (dopamine) (PDA), synthetic melanin-like materials by using 3,4-dihydroxyphenylalanine (l-DOPA) or 5,6-dihydroxyindole(DHI) as precursors, are developed and has attracted considerable interests in recent years [100,101]. PDA also has some favorable properties as expected, such as biocompatibility, reducibility, fluorescence quenching ability, particularly photothermal conversion capability. In 2013, Liu et al. [102] ​confirmed that PDA NPs could be used as PTT agents with high photothermal conversion efficiency (>40%). Since then, a rapid increase of research reports concerning new dopamine-based materials with excellent performance has been witnessed, which have been comprehensively introduced by some earlier reviews [100]. However, there are remaining obstacles that need to be gotten over to further transform these dopamine-based materials from the research to clinical application. A major difficulty lies in the lack of enough understanding of the polymerization mechanism and the exact knowledge of structures of the components of PDA. Moreover, it is in desperate need of investigating the toxicity and immunogenicity of PDA-based PTT agents. It is also noteworthy to fabricate dopamine/PDA with other materials to realize multifunctionalization, a promising direction being pursued in the field of biomedicine [100].

## 2 Categories of nanomedicine for phototheranostic application

Although conventional cancer treatments are broadly used in clinic, they could not completely eradicate tumors meanwhile accompanied by severe side-effects. Besides, the poor aqueous solubility, inadequate selectivity of anticancer drugs, and the occurrence of multidrug resistance after repeated administration limit their therapeutic efficacy. Cancer nanomedicine, regarded as the medical application of nanotechnology to treat cancers, is beneficial to overcome these limitations. Generally, the drugs are usually incorporated into the NPs through distinct mechanisms, including physical encapsulation, absorption or chemical conjugation, and then they can be delivered to the tumor tissues specifically and efficiently. There are several advantages when applying NPs for drug delivery, including unique physicochemical characters, ease of chemical modification to achieve active targeting, controlled release of drugs and enhanced therapeutic effects when compared with small molecular drugs. Nowadays, there has been a wide range of NPs for targeted cancer therapy. By taking advantage of nanomedicine aforementioned, researchers have designed various types of phototherapeutic nanomedicine [[103], [104], [105], [106], [107], [108], [109], [110]]. In this section, the categories of phototheranostic nanomedicine are summarized as well as some latest and representative examples.

### 2.1 Polymer-based NPs

Polymer-based NPs are the most essential NPs, which have been studied as drug carriers for decades. These NPs are usually composed of synthetic polymers, allowing customization of many key characteristics, including molecular weight, hydrophilicity-hydrophobicity, and biodegradability. Meanwhile, various preparation methods, such as nanoprecipitation, electrospray, and emulsification, have also been reported to obtain the polymer-based NPs with desirable properties [103]. Typically, the polymer-based NPs are comprised ​dense matrices with well-known degradation curves, allowing the encapsulation of hydrophobic drugs while further releasing them in targeted locations, making them the excellent candidates for nanophototheranostics delivery [111]. Based on the different chemical structure of building polymers and the morphology of obtained NPs, two types of them (polymeric micelles and polymeric NPs) ​will be discussed as follows.

#### 2.1.1 Polymeric micelles

Polymeric micelles are core-shell NPs self-assembled by amphiphilic polymers, whose formation is facilitated by different molecular interactions, such as hydrophobic interaction, electrostatic interaction, metal complex formation, and hydrogen bonding. Accordingly, polymeric micelles present impressive stability featured by low critical micelle concentration, solid core, and kinetic stability [112]. Besides, through fine-adjusting chemical structures of the micelle-building amphiphilic copolymers, the performance of micelles in drug delivery could be optimized. Furthermore, incorporation of stimuli-responsive cleavable linkers and targeting ligands into the copolymers functionalizes polymeric micelles with smart abilities [113]. Owing to the aforementioned advantages, the micellar formulations carrying PSs and PTT agents have been developed for achieving promising phototherapeutic effect [114]. For example, Pan et al. conjugated IR825-NH_2_ to hydrophilic copolymer methoxy poly (ethylene glycol)-block-poly(l-aspartic acid sodium salt) (PEG-PLD) to offer an amphiphilic polymer PEG-PLD (IR825). Gain from the chemical conjugation of cyanine molecules onto the polymer backbones, PEG-PLD (IR825) micelles achieved an excitingly high drug loading content of about 21% and the minimal IR825 premature release during blood circulation. Finally, the *in vivo* study demonstrated that PEG-PLD (IR825) micelles possessed promising tumor ablation ability during PTT [115]. In addition, PEG-PLD (IR825) micelles displayed polarity-sensitive fluorescence characteristics, which was beneficial for both *in vitro* imaging (Ex: 552 ​nm, Em: ∼610) and *in vivo* NIR fluorescence imaging–guided PTT (Ex: 780 ​nm, Em: 830).

**Fig. 6:**
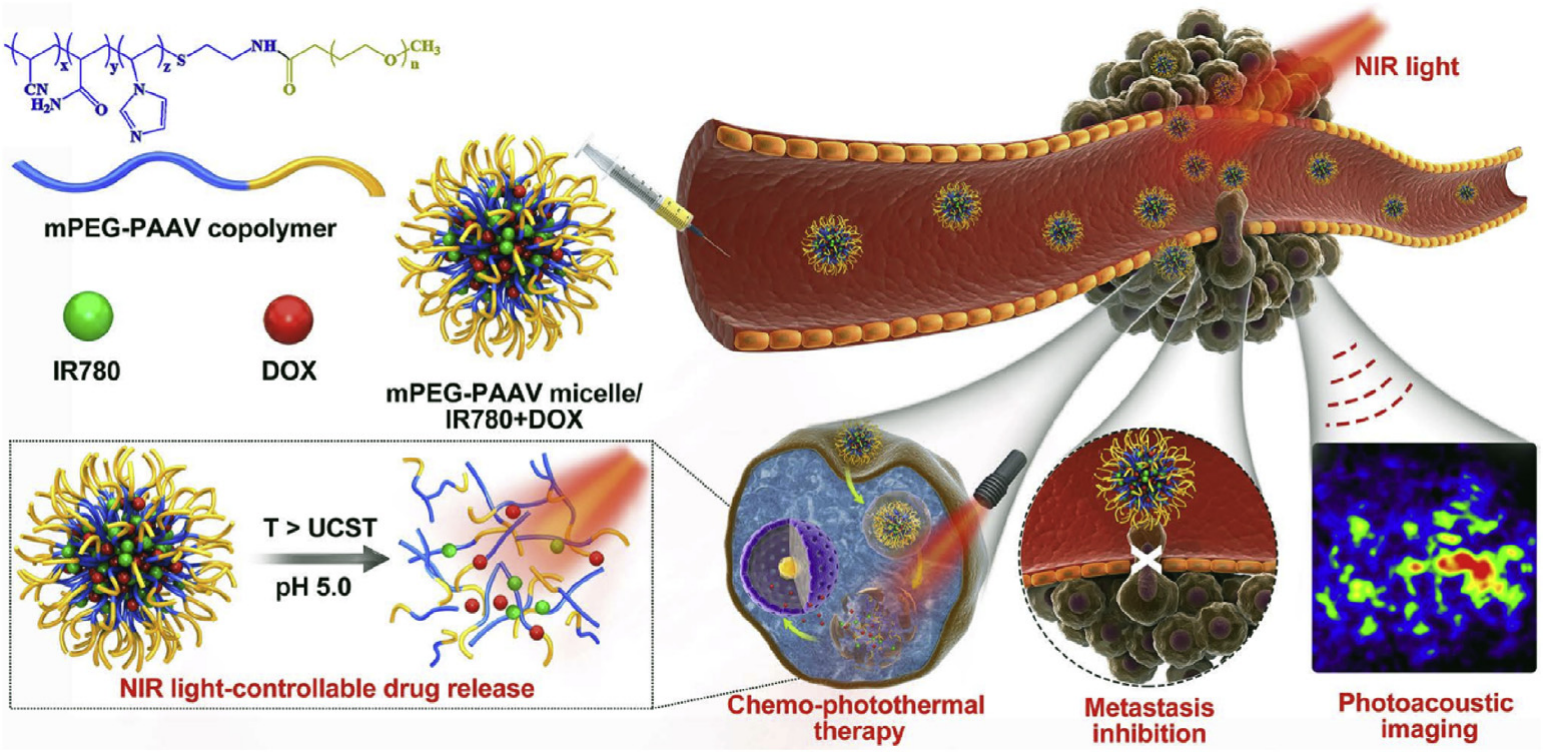
Schematic illustration of cancer chemo/PTT by using mPEG-PAAV micelle encapsulating IR780 and DOX with 808 ​nm laser irradiation [122]. Abbreviations: mPEG-PAAV, poly(ethyleneglycol)-b-poly(acrylamide-co-acrylonitrile-co-vinylimidazole) copolymer; (IR780), (2-[2-[2-Chloro-3-[(1,3-dihydro-3,3-dimethyl-1-propyl-2H-indol-2-ylidene) ethylidene]-1-cyclohexen-1-yl]ethenyl]-3,3-dimethyl-1-propylindolium iodide; DOX, doxorubicin; NIR, near-infrared; UCST, upper critical solution temperature; PTT, photothermal therapy.

With a more in-depth understanding of polymeric micelles, various functionalized micelles have been designed and developed for phototherapy with special purposes. PDT is a kind of oxygen-dependent therapeutic modality, whose efficacy was in positive correlation with oxygen concentration in tumor tissues. However, hypoxia is a hallmark of cancer, which can be further aggravated in the process of PDT, causing compromised photodynamic efficacy [116]. To address this issue, the strategy of tumor oxygenation by polymeric micelles has been proposed and verified to enhance the photodynamic therapeutic efficiency [117,118]. Wang et al. [118] formulated the polymeric micelles via the self-assembly of triblock copolymers of poly(ethylene glycol)-block-poly(acrylic acid)-*block*-polystyrene (PEG-b-PAA-b-PS) followed by chemical conjugation of Hb. In the presence of Hb, an oxygen transporter in the human body, the micelles could generate more ^1^O_2_ and induce more significant photocytotoxicity on HeLa cells than corresponding micelles without Hb. Wang et al. [117] modified the branched polyethyleneimine with perfluoroalkyl groups to possess the micelles of the oxygen-carrying capacity. Based on the results, the chlorin e6 (Ce6, PS) loaded polymeric micelles successfully increased the oxygen level and overcame the hypoxia in C6 glioma cells under oxygen-deficient conditions, leading to the higher therapeutic efficacy than both free Ce6 and control micelles without the perfluoroalkyl groups. Except for tumor oxygenation, the development of stimuli-responsive micelles for controlled release of phototherapeutics and/or anticancer drugs has also attracted much attention [[119], [120], [121], [122], [123]]. Zhao et al. reported dually hypoxia- and ^1^O_2_-responsive polymeric micelles to facilely improve PDT efficacy. In their work, methoxy poly (ethylene glycol)-azobenzene-poly(aspartic acid) copolymer with side-chain modification of imidazole was prepared to form the micelles. The azobenzene collapse could be triggered in the hypoxic tumor microenvironment (TME), causing the detachment of the PEG layer, which finally facilitated cellular uptake of micelles. Afterward, the fast release of Ce6 was observed after micelle disassembly induced by imidazole oxidation [121]. More recently, Wang et al. [123] constructed a novel self-destructive copolymer (PEG-PBC-TKDOX) for the cascade reaction when realizing the stimuli-responsive drug release. The conjugated doxorubicin (DOX) as a hydrophobic domain to facilitate the loading of Ce6 while optimizing the size of micelles in the proper range (50 ​nm). Upon light irradiation, the ROS generated by Ce6 could activate a cascade reaction to release the loaded drugs, which is achieved by grafting the ROS-sensitive pendant thioketal to an aliphatic polycarbonate. They proved that the PEG-PBC-TKDOX/Ce6–based phototheranostic micelles improved efficiency for synergistic PDT/chemotherapy and reduced undesired toxicity. As for PTT-related micelles, thermal-sensitive ones have been widely investigated by taking advantage of the temperature enhancement in the process of treatment. Our group previously synthesized a thermo- and pH-responsive amphiphilic copolymer (mPEG-PAAV) to encapsulate DOX and IR780 for combined chemo/PTT (Fig. 6). As for the mPEG-PAAV, it possessed an upper critical solution temperature (UCST) around 50.8 ​°C at pH 7.4, which decreased significantly at the acidic environment. The heat generated during PTT as well as the acidic TME, as dual stimuli, induced the disassembly of micelles to the rapid release of DOX for chemotherapy. Accordingly, the high chemo/PTT synergetic efficacy was achieved both *in vitro* and *in vivo* [122]. It is also noteworthy that mPEG-PAAV/IR780+DOX micelle could serve as PA imaging agents to monitor the morphology and microvascular distribution of tumor tissues, finally guiding the micelles-mediated chemo-PTT.

**Fig. 7:**
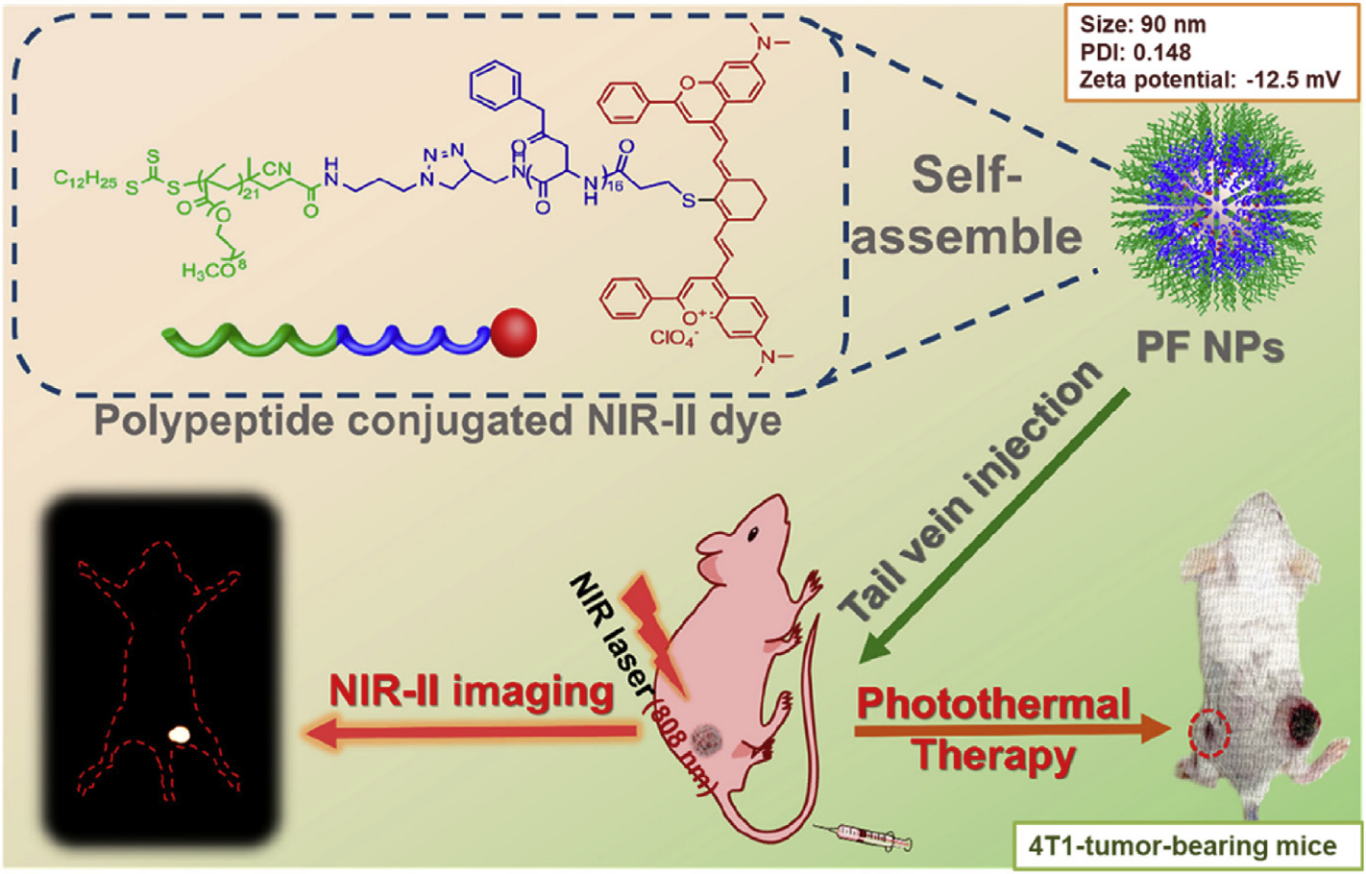
Schematic illustration of the PF NPs-assisted NIR II image–guided PTT by PF NPs [126]. PF NPs, fluorophore nanoparticles; PTT, photothermal therapy; NIR near-infrared.

Furthermore, some other polymeric micelles with well-designed formations have also been studied and reported. For example, Liu et al. [124] used the PS (5,10,15,20-tetrakis (4-carboxyphenyl) porphyrin (TCPP)), as the tetrafunctional cross-linker to induce the cross-link of the shell of micelles, to enhance drug loading content and their stability. Besides, the TCPP bridges also possessed manganese II (Mn^2+^) chelating sites promising for T1-weighted magnetic resonance imaging (MRI). Yang et al. [125] developed a kind of NIR dyes-conjugated polymer HRGP-IR with oxidative stress amplifying function and self-assemble them into micelles for combinational oxidative phototherapy. In the acidic intracellular compartment, the hydrogen peroxide–generating compounds cinnamaldehyde could be released rapidly, allowing the progress of Fenton reaction to produce highly toxic hydroxyl radical, inducing dramatically cell death. Meanwhile, the conjugated IR820 is responsible for fluorescence and PA imaging, light-to-heat conversion and ROS generation, further improving the therapeutic efficiency. Recently, Li et al. [126] reported an excellent photothermal ablation outcome on 4T1 tumor-bearing BALB/c mice with a low dose of NIR-II dye and low laser power, in which the micelles ​self-assembled from facile macromolecular fluorophore(PF) played an essential role (Fig. 7). A small-molecule NIR II dye (Flav7) was chemically modified to the terminal of an amphiphilic polypeptide in the first step followed by micelles formation. The PF NPs then displayed strong photothermal stability, high photothermal conversion efficiency, and minimized dark cytotoxicity. Upon laser irradiation, severe photothermal toxicity could be induced both *in vitro* and *in vivo*. More importantly, the PF NPs served as fluorescent probes to visualize and feature the tumors through a NIR-II fluorescence imaging system because of the presence of Flav7, enhancing the image quality and minimizing the interference from inherent tissue autofluorescence.

#### 2.1.2 Polymeric NPs

Different from micelles, polymeric NPs are recognized as solid NPs comprised hydrophobic polymers. So far, a large amount of polymers has been applied to formulate the NPs, among which PLGA occupies an important position. PLGA, which was approved by FDA, has been exploited as an excellent biocompatible, biodegradable, and non-toxic polymer with various applications in tissue engineering, medical and surgical devices, as well as drug delivery since the early 1970s. By adjusting the monomer ratio of lactic acid to glycolic acid, the physicochemical properties of PLGA, including glass transition temperature, density, crystallinity, and viscosity, could be tailored to meet different requirements [127]. Thus, researchers have encapsulated phototherapeutics into PLGA NPs to develop efficient phototheranostic nanomedicine [[128], [129], [130]]. Shen et al. [129] designed a bioinspired, ICG and DOX coloaded, PLGA-based theranostic nanoplatform IDPNsquery, which is modified with bovine serum albumin, for fluorescence/PA imaging–guided cancer chemotherapy/PTT. The IDPNs displayed an impressive photothermal effect and heat controlled release behavior of DOX. In addition, their inhibition rate against EMT-6 tumors was high up to 95.6%.

**Fig. 8:**
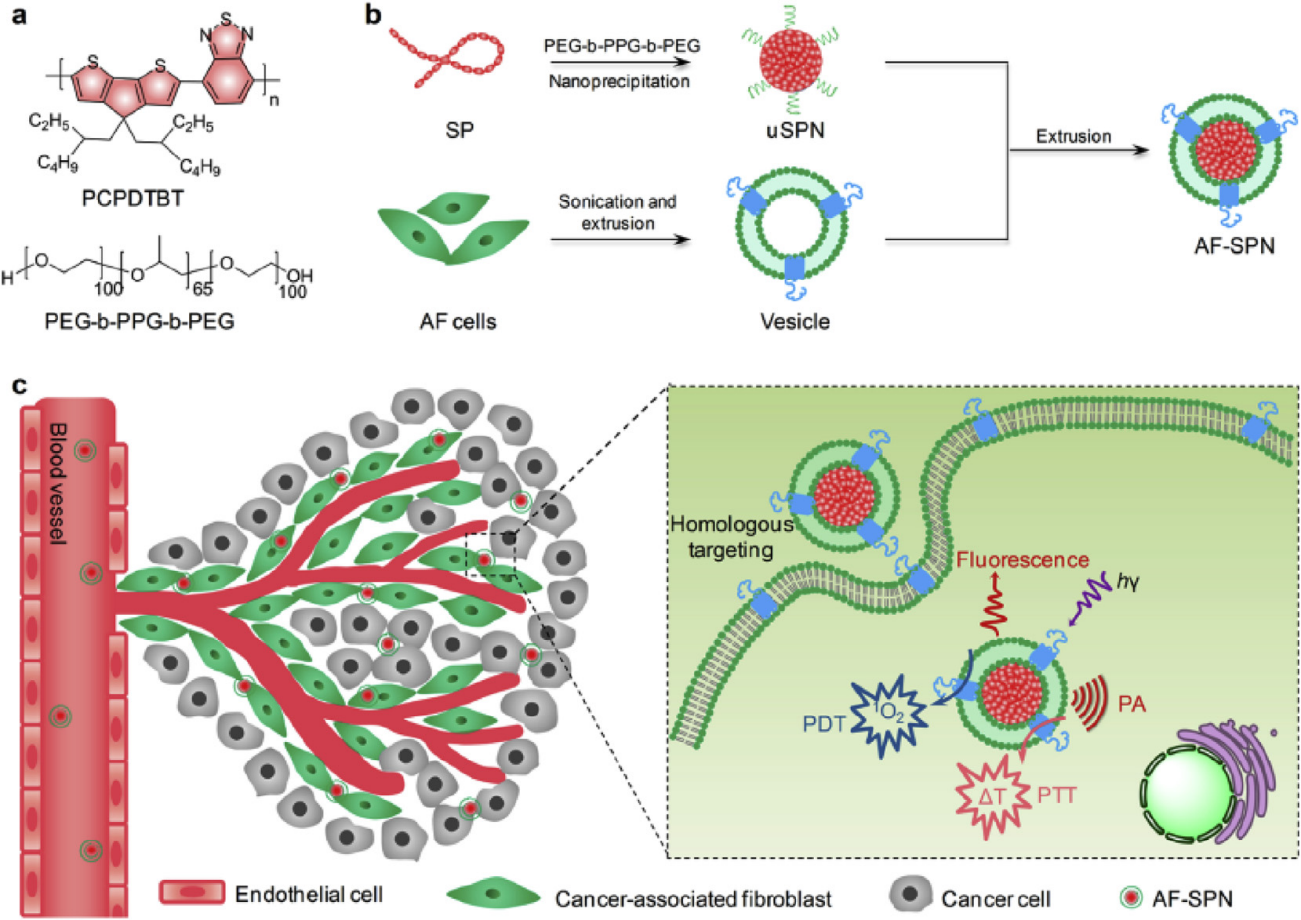
Schematic illustration of organic multimodal phototheranostic nanosystems, AF-SPN, for multimodal imaging–guided cancer phototherapy, including chemical structures of various components (a), preparation procedure of AF-SPN (b), and related mechanism under AF-SPN mediated diagnosis and phototherapy (c) [137]. AF-SPN , activated fibroblasts semiconducting polymer nanoparticle.

Recently, a novel type of organic optical nanostructures, semiconducting polymer nanoparticles (SPNs), has also been developed for biomedical applications [131,132]. SPNs are usually comprised semiconducting polymers (SPs), showing advantages including organic and biologically inert, high absorption coefficients, and preferred photostability. Owing to their excellent optical properties, SPNs have been applied in cancer phototherapy. For example, Zhou et al. [133] prepared the polyaniline NPs coated with F127 to obtain the F127-modified PANPs, named F-PANPs. Based on their results, F-PANPs displayed an impressive molar extinction coefficient (8.95 ​× ​10^8^ ​M^−1^ ​cm^−1^), as well as high NIR photothermal conversion efficiency (48.5%). Yang et al. [134] demonstrated that polypyrrole, another kind of SPs, can be used for photothermal tumor ablation at low laser power density. However, there is an obvious drawback for the first generation of SPNs, which is their weak and broad absorption in the whole NIR region. To overcome this issue, many attempts have been made through improving the chemical structure of SPs with the purpose of sharping NIR absorbance peaks [96,135,136]. For example, Guo et al. [96] designed and synthesized a novel donor-acceptor typed SP (PorCP), with porphyrin in the polymer backbone, showing the absorption peak at 799 ​nm. The formed SPNs by PorCPs displayed an impressive photothermal conversion efficiency (63.8%), which was much higher than that of clinically applied PTT agents. Later, Jiang et al. [135] reported the first organic photothermal nanoagent (SPNI II) with dual-peak absorption in both NIR windows and its phototherapeutic-related properties were comprehensively investigated. Except for PTT, SPs can also activate oxygen to ROS upon light irradiation. As an extension of previous work, Li et al. [137] ​further developed the organic multimodal phototheranostic nanosystems comprised SPN coated with membranes of activated fibroblasts, (AF-SPN), for multimodal imaging–guided cancer phototherapy. As shown in Fig. 8, the AF-SPN displayed a specific targeting effect on cancer-associated fibroblasts, improving the tumor accumulation of NPs in the tumor tissues. Besides, combined PDT/PTT mediated by AF-SPN led to a dramatic antitumor efficacy. Moreover, Tang et al. [138] attempted to encapsulate or conjugate the PS to SPNs to amplify the generation of ROS upon light irradiation. In these polymeric NPs, SPs efficiently absorbed and transferred the energy of light to PS, amplifying the generation of ROS.

### 2.2 Liposomes

Liposomes, one of the first-generation nanomedicine for clinical applications, were primarily described in 1965. These spherical NPs are formed from single or multiple lipid bilayers, comprising an aqueous core and a vesicle shell. Owing to their unique structure, liposomes can load water-soluble drugs into their aqueous cavity and hydrophobic ones in their lipid shell simultaneously, which prevents the agents from degradation. Besides, the superiorities of liposomes, such as high stability in physiological conditions and controllable drug release, endow them with better performance in pharmacokinetics and biodistribution of cargoes [139]. Therefore, liposomes have become promising nanosystems to deliver ​phototherapeutics for phototherapy [140,141]. Zhang et al. [140] constructed the bioinspired melanin-based PEGylated nanoliposomes (Lip-Mel) as theranostic agents for dual imaging (PA imaging and T1-weighted MRI)–guided cancer PTT. The efficient entrapment of melanin into Lip-Mel relieved the toxicity of free melanin to normal tissues although improved the PTT efficiency of the loaded melanin at tumor sites.

**Fig. 9:**
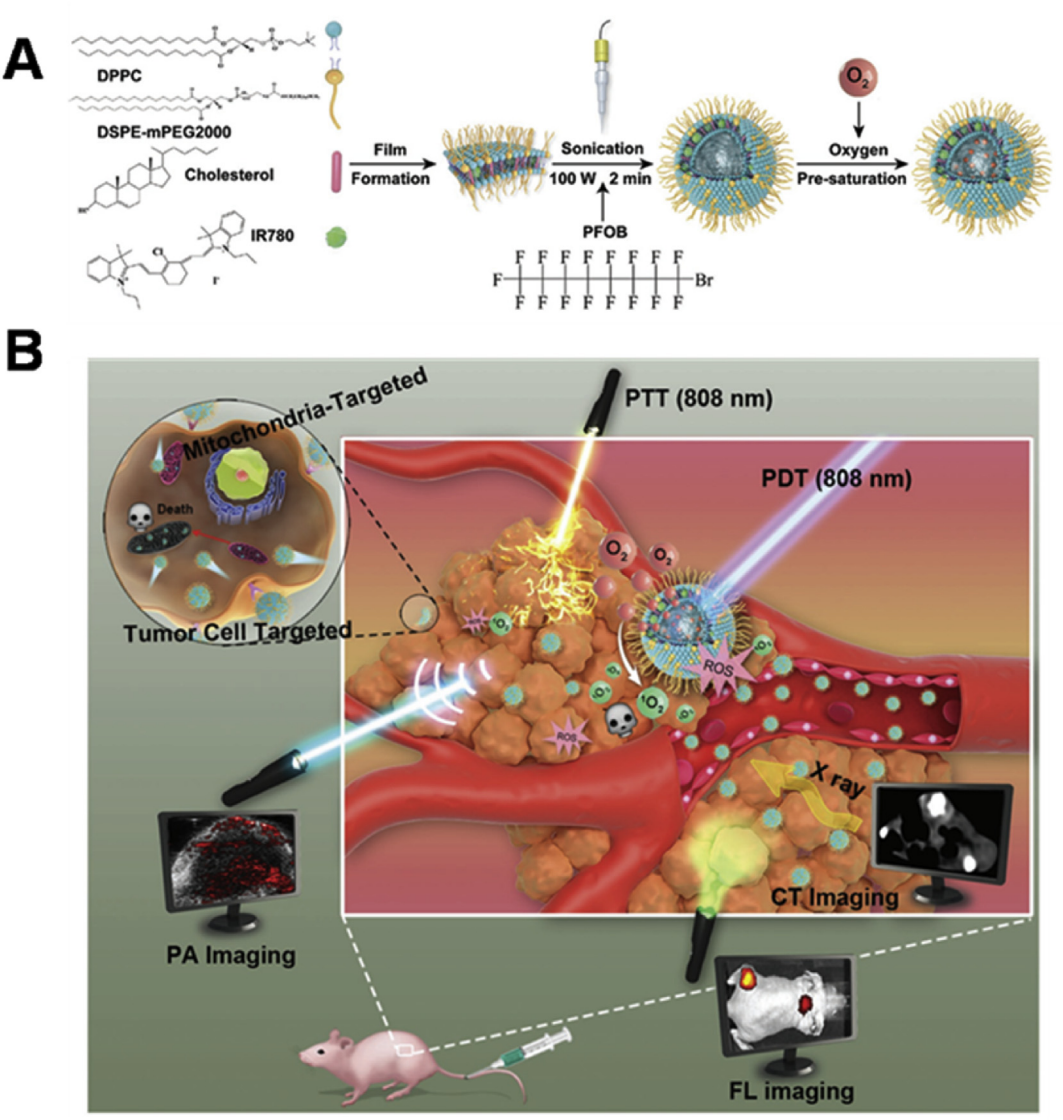
(A) Illustration of the formation of PFOB@LIP-IR780. (B) Schematic illustration of the multifunctional ‘Nano-RBCs’ when performing the triple imaging–guided cancer PDT/PTT in the presence of oxygen supplier PFOB [142]. PFOB, perﬂuorooctyl bromide; PDT, photodynamic therapy; PTT, photothermal therapy.

As aforementioned, the hypoxic TME usually limits PDT efficacy, thus, it is necessary to develop more potential strategies to overcome this drawback, such as tumor oxygenation. As shown in Fig. 9, Zhang et al. [142] prepared the perﬂuorooctyl bromide (PFOB)–based liposomes for oxygen transportation to tumor tissues. Through alleviating the tumor hypoxia, the photodynamic action and photothermal conversion capacity of IR780, which was anchored in the lipid layers, could be significantly amplified. Around the same time, Sheng et al. [143] also developed the liposomes with the same formula encapsulating ICG to enhance PDT/PTT synergistic therapy by taking advantage of PFOB's oxygen-carrying capacity. In addition, calcium peroxide (CaO_2_) has been used as an O_2_-generating material to allay hypoxia. Liu et al. [144] incorporated CaO_2_ into the lipid layers of liposomes to obtain an O_2_ self-sufficient ingenious liposome nanoplatform (LipoMB/CaO_2_) for PDT under dual-stage light irradiation, which proved to be a successful attempt for PDT against hypoxic tumor.

Except for the tumor oxygenation, incorporating inhibitors into the nanosystems is another promising approach to overcome the negative influence of tumor hypoxia on PDT. It has been reported that metformin, a respiration-related inhibitor with the principle of inhibiting the function of complex I in the mitochondrial electron transport chain, could efficiently reduce the tumor oxygen consumption [145]. Song et al. [146] coloaded hydrophilic hypoglycemic agent metformin and modified hydrophobic PS HCe6 into the liposomes formulated by DSPE-PEG, DPPC, and cholesterol, to enhance PDT efficacy via modulating tumor hypoxia. As another interesting sample, Broekgaarden et al. [147] prepared the PEGylated cationic liposomes containing the ZnPc in their lipid bilayers and acriflavine (ACF) in their aqueous core for PDT against human epidermoid carcinoma. Accordingly, the PDT efficacy would be compromised because of the hypoxia TME. Besides, the activation of hypoxia-inducible factor 1 (HIF-1) survival pathway could also lead to the PDT resistance. ACF, as the HIF-1 inhibitor, could prevent HIF-1α/HIF-1β dimerization by binding to the dimerization domain of HIF-1α, hence inhibiting tumor cell survival pathways [148]. Based on their results, inhibition of HIF-1 by ACF enhanced the PDT efficacy under hypoxic conditions [147].

Although the liposomes were capable of enhancing the localization of drugs at the tumor tissues, there will be insufficient therapeutic efficacy once the drugs are not released timely. To realize the controlled drug release, Li et al. [149] formed a light-sensitive liposome (Her2-I&D-LSL) using a specially designed phospholipid (PLsPC) and a hydrophobically modified PS (ICG-ODA). Besides, anticancer drug DOX was also encapsulated into it. Upon NIR light irradiation, Her2-I&D-LSL could generate a large amount of ROS for efficient PDT. Furthermore, the ROS could disturb the integrity of liposomes, triggering the release of DOX. Based on their results, the ROS generation and DOX release were controlled by tuning the NIR light and ICG-ODA loading content in liposomes. He et al. [150] designed photothermally sensitive nanoliposomes to encapsulate sorafenib (SF) and ICG to solve the problems of SF-based treatment in advanced hepatocellular carcinoma. The heat generated in the process of PTT could induce the damage of liposomal structural integrity to release the SF immediately.

### 2.3 Nanogels

Hydrogels are three-dimensional polymeric networks and capable of absorbing high amounts of water or biological ﬂuids because of their hydrophilic structures [[151], [152], [153], [154], [155]]. Nanogels are nanosized hydrogel particles formulated by physical or chemical cross-linked polymer networks. In the past decades, they have been applied in various biological fields, including drug delivery, tissue engineering, biosensors and so on. Particularly, the nanogels have also been used for phototherapy by taking the aforementioned advantages of nanomedicine and their impressive features (e.g. water solubility and high water level). The nanogels prepared by polyacrylamide (PAA) is an illustration of nanogels used for cancer PDT. The nearly neutral (zeta potential) surface of PAA-based nanogels could prevent the rapid removal of themselves by macrophages. Moreover, low protein adherence and high water level could further reduce their opsonization in blood circulation, rendering them “underground” to the macrophages [156,157]. Gao et al. [157] used a modified emulsion approach to encapsulate the meta-tetra (hydroxyphenyl) chlorine (mTHPC) into PAA nanogels for cancer PDT. Their ultrasmall size not only protects themselves from being evaded by the reticuloendothelial system (RES) but also empowers the more rapid diffusion of ROS out of the nanogels. Moreover, the nanogels can be removed through renal clearance, reducing the risk of drug accumulation in the human body. Besides, the PS could also be conjugated to PAA to evade the premature release [158,159]. For example, Kopelman's group developed a multifunctional nanosystem using amine-functionalized PAA nanogels. The interesting design contains incorporating primary amino groups and cross-linkers into nanogels during their polymerization. Meanwhile, PS and fluorescent dyes were encapsulated into nanogels, and PEG and tumor-targeting ligands were further modified onto their surface. The obtained NPs can be transported efficiently into tumor cells accompanied by obvious intracellular fluorescence. In addition, through using the laser at a proper wavelength for irradiation, they induced obvious but selective destruction to the cancer cells inside the irradiated fields [159].

Actually, instead of organic PSs, some inorganic materials such as TiO_2_, titania, and GNRs can also be loaded into nanogels for PDT [160,161]. Kirakci et al. [160] first ​prepared a luminescent complex, Na_2_[Mo_6_I_8_(1-OOC-1,7-closo-C_2_B_10_H_11_)_6_], showing a high fluorescence quantum yield of 93% and a high ^1^O_2_ generation efficiency of 70%. Through further blending with β-cyclodextrin, the monodisperse nanogels have been obtained for potential applications in cancer PDT.

In addition, the PTT agents–loaded nanogels have also been developed rapidly, achieving effective therapeutic efficiency. A chitosan derivative containing polyaniline in side chains, synthesized by Siao et al., could self-assemble into micelles and then transform into nanogels facilitated by a pH alteration, which could play a role as nanoscaled heating sources in selectively killing cancer cells within the targeting area. In addition, owing to the excellent spatial stability of nanogels within a solid tumor, the leakage of nanogels from original injection sites could be minimized even with repeated NIR irradiation, resulting in the enhanced therapeutic efficacy when compared with the controlled hollow gold nanospheres [162].

**Fig. 10:**
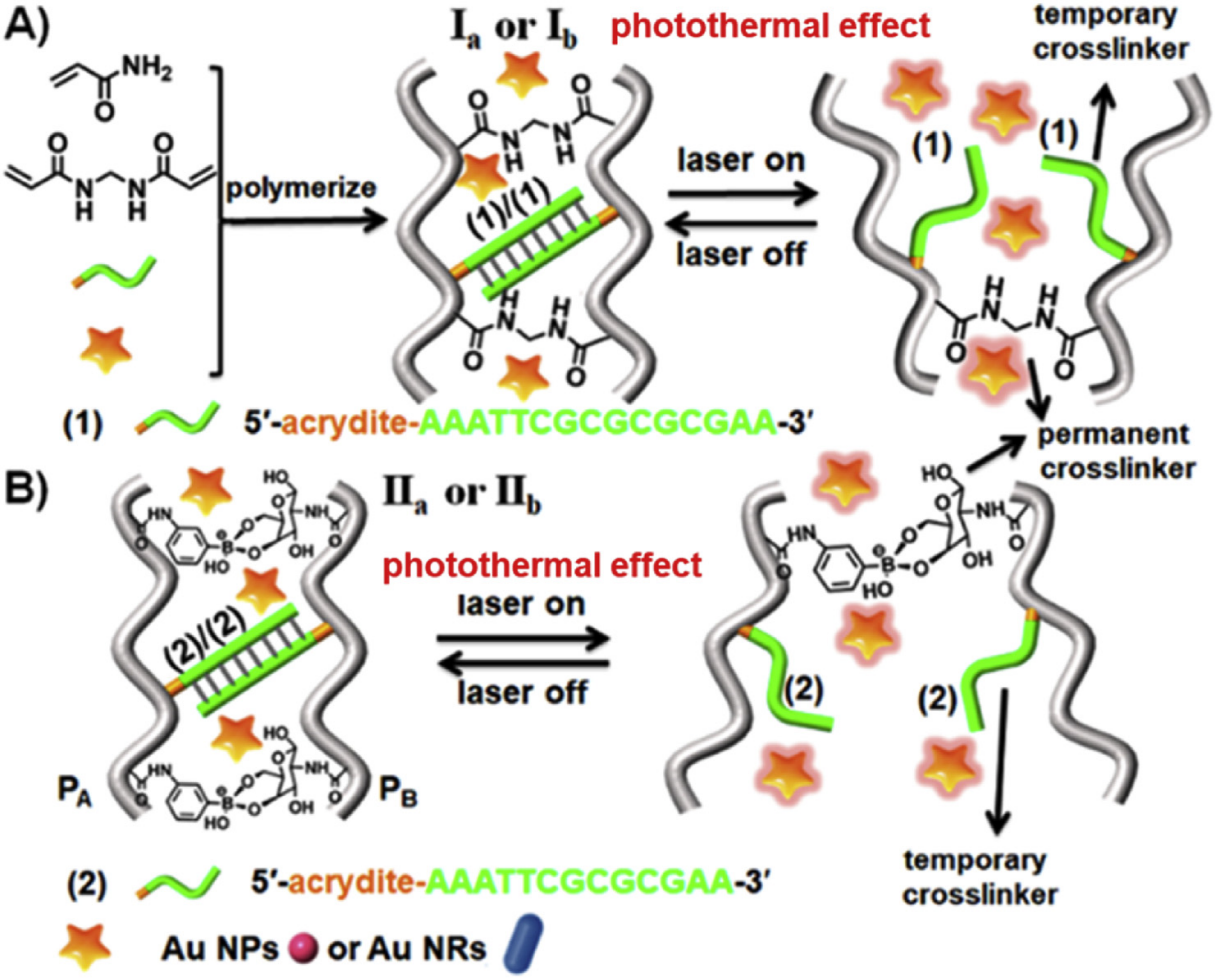
Thermoresponsive stiffness control of nanogels in the presence of GNPs (Ia and IIa)and GNRs (Ib and IIb), which is cross-linked by bis-acrylamide and nucleic acid duplexes (1)/(1) (A) or boronate ester-glucosamine and nucleic acid duplexes (2)/(2) (B), respectively [166]. GNRs, gold nanorods; GNPs, gold nanoparticles.

More recently, in situ formed thermosensitive nanogels encapsulating PTT agents have shown the great potential of achieving simultaneously NIR-activated phototherapy and drug release [[163], [164], [165]]. For example, Liu et al. [163] developed photothermal network based thermosensitive hydrogel (PNT-gel) loading ICG for combinatorial PDT/PTT. Interestingly, the supramolecular cross-linking SPs were also incorporated to generate the photothermal network. The PNT-gel exhibits a reversible gel-to-sol UCST around 40 ​°C and the release of ICG could be hence controlled through a NIR-induced photothermal-mediated gelsol transition. Another kind of thermosensitive nanogels was prepared by Wang et al. [166] very lately. As displayed in Fig. 10, PAA nanogels were permanently cross-linked by bis-acrylamide or boronate ester-glucosamine. Meanwhile, temporary cross-linking was introduced into the nanogels by nucleic acid duplexes. In the presence of GNPs or GNRs in the nanogels, the dehybridization of the DNA duplexes occurred upon laser irradiation ​because of the photothermal effect of these metallic NPs, leading to the formation of nanogels with lower stiffness. Through changing the irradiation light, the nanogels are switched between low- and high-stiffness states in a reversible modal, which is beneficial to develop shape-memory nanogels, self-healing soft materials, phototherapeutic nanosystems with controllable drug release behavior.

### 2.4 Dendrimers

Dendrimers, which are three-dimensional, unimolecular, highly branched monodispersed macromolecules [167,168], can also be used for effective drug delivery and diagnosis. They contain an original core from which branches begin to extend, as well as the terminal functional groups on their surface. Usually, the exterior surface of dendrimers is functionalized by water-soluble components for improving their water solubility, whereas the hydrophobic inner core and branches are used to encapsulate the drugs. As good candidates, dendrimers could also be used as nanocarriers to deliver the phototherapeutic agents [169]. Kojima et al. [170] synthesized the PEGylated dendrimers for encapsulating PSs including RB and protoporphyrin IX (PpIX) for PDT. Two PEG-attached dendrimers are being prepared by the authors, the PAMAM dendrimer of G4 (PEG-PAMAM) and the PPI dendrimer of G5 (PEG-PPI). The results demonstrated that relatively fewer PpIX molecules were encapsulated by both dendrimers than RB, but the PpIX involved complexes were more stable under physiological conditions. Moreover, the complex PEG-PPI/PpIX exhibited more significant cytotoxicity on tumor cells compared with free PpIX. Another interesting dendrimer has been developed by Aida and Jiang [171] and Nishiyama et al. [172] ​for efficient PDT. In such cases, the dendrimer is used as the therapeutics by themselves. Briefly, the dendrimer porphyrin, containing a porphyrin as the inner core, is surrounded by a framework of some aryl ether dendrites. They can transport energy from light irradiation to the porphyrin core and produce highly toxic ^1^O_2_ . The clinical applicability of Pcs is undesirable because of their weak water solubility and poor targeting effect on cancer cells. Taratula et al. [90] developed an effective Nc-contained theranostic nanoplatform for NIR fluorescence imaging–guided PDT/PTT. In this case, silicon Nc (SiNc) was loaded into the inner sites of the G5 PPI dendrimer, which was further coated with PEG. Because that the PPI dendrimer has hydrophobic domains, the SiNcs were stably encapsulated with negligible aggregation. Furthermore, their functions of fluorescence imaging, PDT, and PTT were preserved for cancer diagnosis and treatment.

### 2.5 Non-biodegradable NPs

Non-biodegradable NPs are a large class of NPs which could not degrade rapidly in biological systems. However, owing to their impressive optical properties and easy adjustment in their morphology, non-biodegradable NPs–based multifunctional theranostic nanosystems have also obtained much attention recently in the field of phototherapy.

#### 2.5.1 Silica NPs

Silica is one of the major components of sand, verified to be compatible in biological systems. In the past few decades, a large amount of silica-based NPs has been prepared for different applications, including phototherapy. As for PDT, silica nanostructures, such as silica NPs, organically modified silica (ORMOSIL), and MSNs are representative ones commonly studied. These NPs are good candidates for phototherapy because of their excellent properties, such as chemical inertness, porosity, and easily controlled surface chemistry undergoing preparation [173].

In 2003, Yan et al. [174] used silica NPs to embed PS mesometatetra (hydroxyphenyl) chlorine (m-THPC) via a modified Stöber solgel approach and investigated their ^1^O_2_ generation and pH-dependent optical characters. Later, the same group reported the MB-loaded ORMOSIL NPs for efficient PDT [175]. In another study, to prevent the drug release during systemic circulation, Ohulchanskyy et al. [176] covalently conjugated the PS molecules onto the surface of ORMOSIL NPs. These NPs could be significantly internalized by cancer cells *in vitro* and exhibited impressive phototoxicity during PDT.

**Fig. 11:**
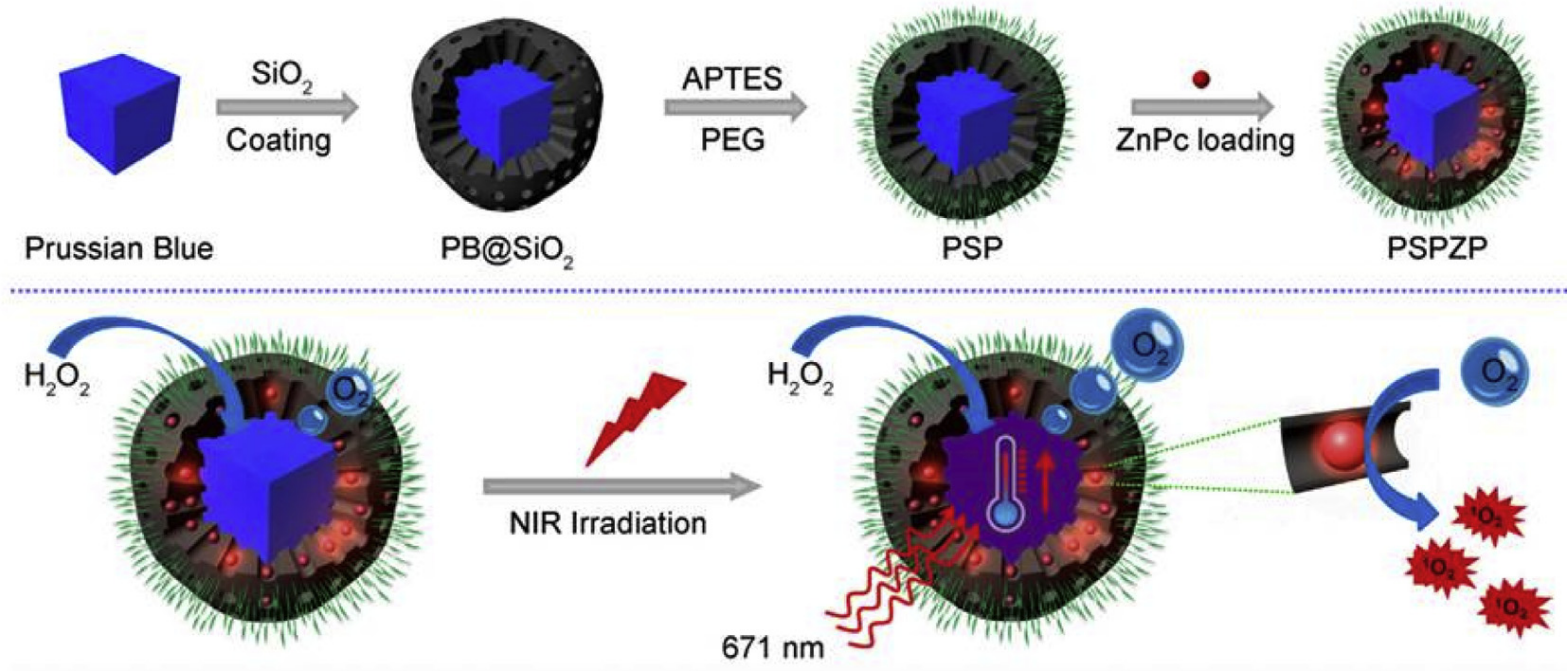
Schematic illustration of the preparation procedure and photoenhanced therapy of the PSP-ZnPc (PSPZP) NCs [183].

MSNs, another important kind of silica NPs, and their derivatives have been utilized to encapsulate PSs, etc. for PDT and other applications, by taking their unique advantages of large surface area, high pore volume, and relatively uniform pore size [[177], [178], [179], [180], [181]]. Wong et al. [182] conjugated an acid-cleavable acetal-linked ZnPc dimer to alkyne-modified MSNs. The fluorescence emission and ^1^O_2_ generation of dimeric ZnPcs inside the mesopores were significantly self-quenched because of their close proximity. Once incubated in the acidic tumor environment, this ZnPc-encapsulated nanosystem would be activated, proved by enhanced fluorescence emission and ^1^O_2_ production. Prussian blue (PB) presented efficient photothermal conversion, making it a promising PTT agent. Besides, PB could also catalyze the hydrogen peroxide to oxygen rapidly. Based on the special properties of PB, Wang et al. [183] developed a phototherapeutic nanosystem PSP-ZnPc (PSPZP) NCs for augmented phototherapy (Fig. 11). Briefly, PB was coated with mesoporous silica first ​while loading ZnPc into the mesopores. To enhance the physiological stability of NPs, PEG chains were further modified onto their surface. Upon 671 ​nm laser irradiation, the local temperature increased significantly and the oxygen concentration in tumor tissues because of the presence of PB core. Besides, the photocytotoxicity of ZnPc was also activated in the presence of light, which could be further amplified by the mass-produced oxygen in situ. Based on the results, the PSP-ZnPc (PSPZP) NCs could achieve promising phototherapeutic efficacy in tumor tissues with the hypoxic condition.

#### 2.5.2 Metallic NPs

Metallic NPs exhibit a large number of promising features and have been widely applied in the field of biomedicine. As mentioned in Section 1.2.2.1, some metallic NPs with excellent photothermal conversion ability could be used for efficient PTT. They also possess fascinating advantages when combined with PS for PDT. GNPs are well-known metallic NPs because of their chemical inertness and minimum acute cytotoxicity. For the GNPs-based nanoagents, PS could be conjugated/loaded on the surface of the GNPs. Because GNPs can be optimized to a larger specific surface area, a high dose of PS can be loaded on their surface [184]. Hone et al. synthesized ultrasmall GNPs with a diameter of 2–4 ​nm whereas a ZnPc derivative containing the mercaptoundecyl group was modified onto the surface of GNPs via stable S–Au bonds. Those ZnPc-modified GNPs were able to generate more ^1^O_2_ upon light illumination as compared with free ZnPc [185]. Recently, Haimov et al. [186] attempted to conjugated mTHPC to GNPs through a linker. The resultant AuNP-mTHPC is a stable, soluble compound showing promising PDT effect against SH-SY5Y human neuroblastoma cells. Another type of gold-based NPs, GNRs, has also been used as PS carriers for PDT after proper surface modification. Tham et al. [[187], [188], [189]] anchored silylated ZnPc onto silica-coated GNR and further grafted with hyaluronic acid (HA). Independent LSPR of GNRs and ^1^O_2_ production of anchored ZnPc guaranteed the phototherapeutic efficacy of obtained GNRs-Si-ZnPc. Attributable to the CD44 targeting effect of HA, the final NPs could be uptaken by CD44-overexpressed cancer cells for synergistic PDT/PTT.

It is also worth mentioning that ^1^O_2_ can also be generated by the metallic NPs themselves in absence of PSs, which was first observed by Vankayala et al. [190] ​in 2011. They observed phosphorescence emission of ^1^O_2_ around 1268 ​nm when metal NPs (Ag, Pt, and Au) were undergoing light irradiation, indicating the potential of using metallic NPs as PSs directly. More recently, Long et al. [191] revealed that surface facet plays an important role in tailoring the ^1^O_2_ production process on metal nanocrystals. By investigating the photoactivity of single-facet Pd nanocrystals, they observed that Pd (100) performed better PDT when killing HeLa cells than Pd (111).

#### 2.5.3 Magnetic nanoparticles

Magnetic nanoparticles (MNPs) are comprehensively studied by researchers in the past few decades, in the aspect of their inherent MRI contrast, magnetic hyperthermia functions, and the capability to target delivery of drugs under the magnetic field [192,193]. Kim et al. [194] designed multifunctional AHP@MNPs containing Fe_3_O_4_ NPs and PS modified HA photosensitizer conjugated hyaluronic acid (AHP) for cancer diagnosis and treatment. The AHP@ MNPs showed improved water solubility, efficient heat and high ^1^O_2_ generation efficiency upon irradiation by dual-energy sources (magnetic and laser). Besides, the AHP@MNPs can be uptaken by cancer cells via CD44 receptor–mediated endocytosis that further promoted the synergistic therapeutic efficacy of AHP@MNPs against cancer cells.

Besides, MNPs were also used to facilitate PS to accumulate in tumor tissues under the magnetic field. Li ​et al. [195] prepared the PEGylated iron oxide nanoclusters (IONCs) to load Ce6. The obtained IONCPEG-Ce6 could be used for efficient cancer diagnosis and therapy by taking the advantages of IONCs-mediated MRI and tumor targeting, and Ce6-induced fluorescence imaging and PDT. In their work, the strong magnetic field attracted IONC-PEG-Ce6 to tumor tissues, showing strong intratumoral fluorescence and magnetic resonance signals. Besides, *in vivo* tumor suppression experiments by using IONC-PEG-Ce6 under magnetic field and laser irradiation further displayed great therapeutic efficacy of this nanoplatform.

#### 2.5.4 Semiconductor QDs

Semiconductor QDs have unique optical properties and their absorbance could be accurately tailored from the UV to NIR region by changing the size in the range of several nanometers. Furthermore, the surface of QDs can be modified to enhance their water solubility and biocompatibility. Most importantly, owing to the presence of large transition dipole moment for QDs, they could be used as energy donors for energy transfer toward ​other acceptors, leading to the exciting photodynamic action for PDT [196,197]. For the QDs themselves, the ^1^O_2_ generation efficiency was relatively low when compared with traditional PSs, but the researchers verified that an enhanced energy transfer efficiency (>75%) could be achieved by using the fluorescence resonance energy transfer (FRET) from QDs to PSs [198].

**Fig. 12:**
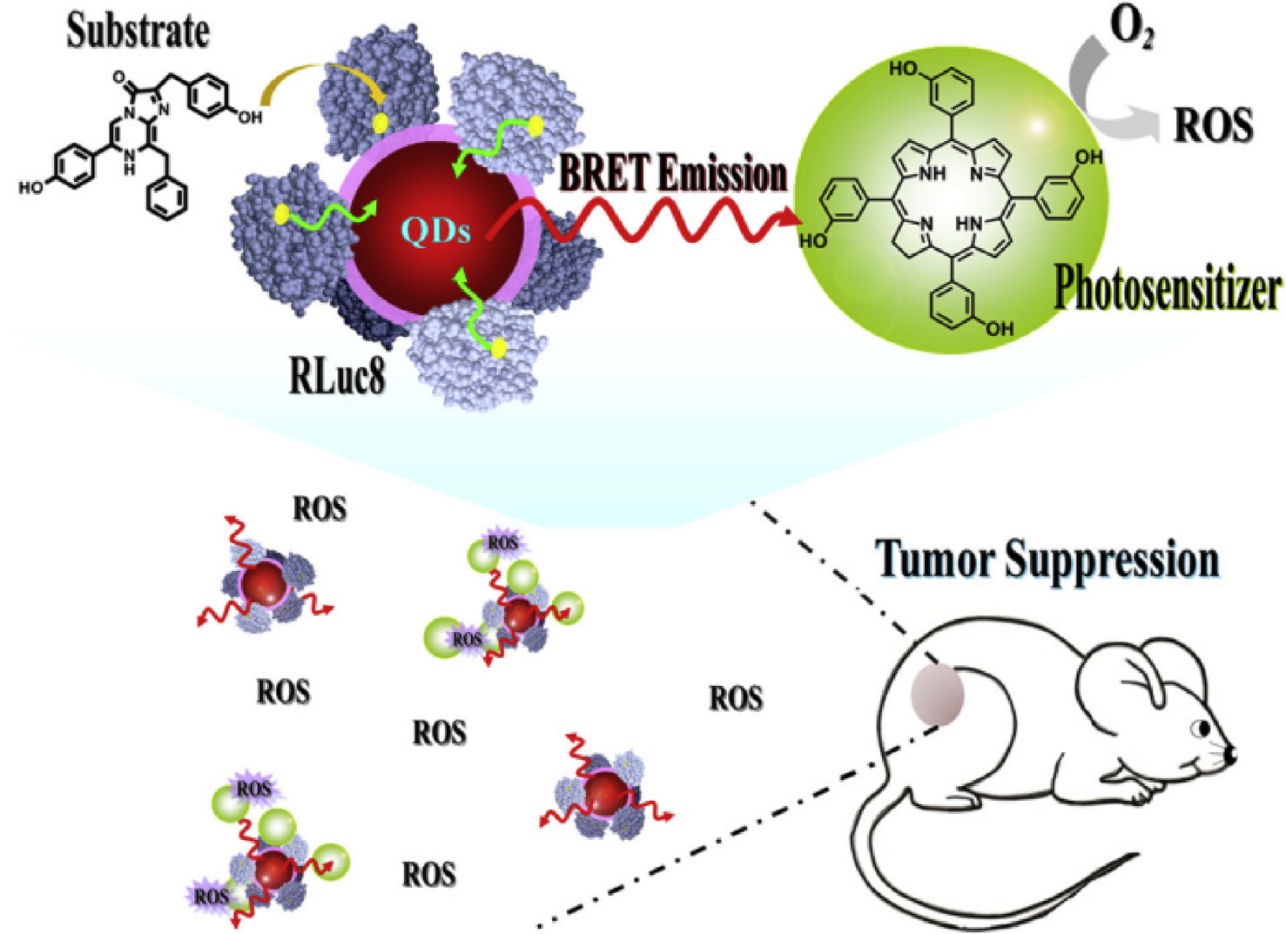
Schematic illustration of RLuc8-immobilized QDs-655 for bioluminescence resonance energy transfer (BRET)–based PDT [199]. PDT, photodynamic therapy; QDs, quantum dots.

There are some other interesting QDs-related projects that have been conducted by different groups to overcome the limitations of PDT. For instance, Hsu et al. [199] developed a strategy to conquer the limitation of light penetration during PDT. As shown in Fig. 12, they developed a Renilla luciferase–immobilized QDs (QD-RLuc8) conjugate. In the presence of coelenterazine (the substrate of RLuc8), the released energy from RLuc8 could be transferred to QDs via BRET, resulting in the autoillumination of QD-RLuc8 conjugate at a wavelength of 655 ​nm. Subsequently, this photon emitted from QD-RLuc8 could further trigger the Foscan-encapsulated micelles to generate ROS and kill the cancer cells. However, the possible toxicity of heavy metal elements–based QD is still one of the obstacles for their clinical translation.

#### 2.5.5 Nanocarbons

**Fig. 13:**
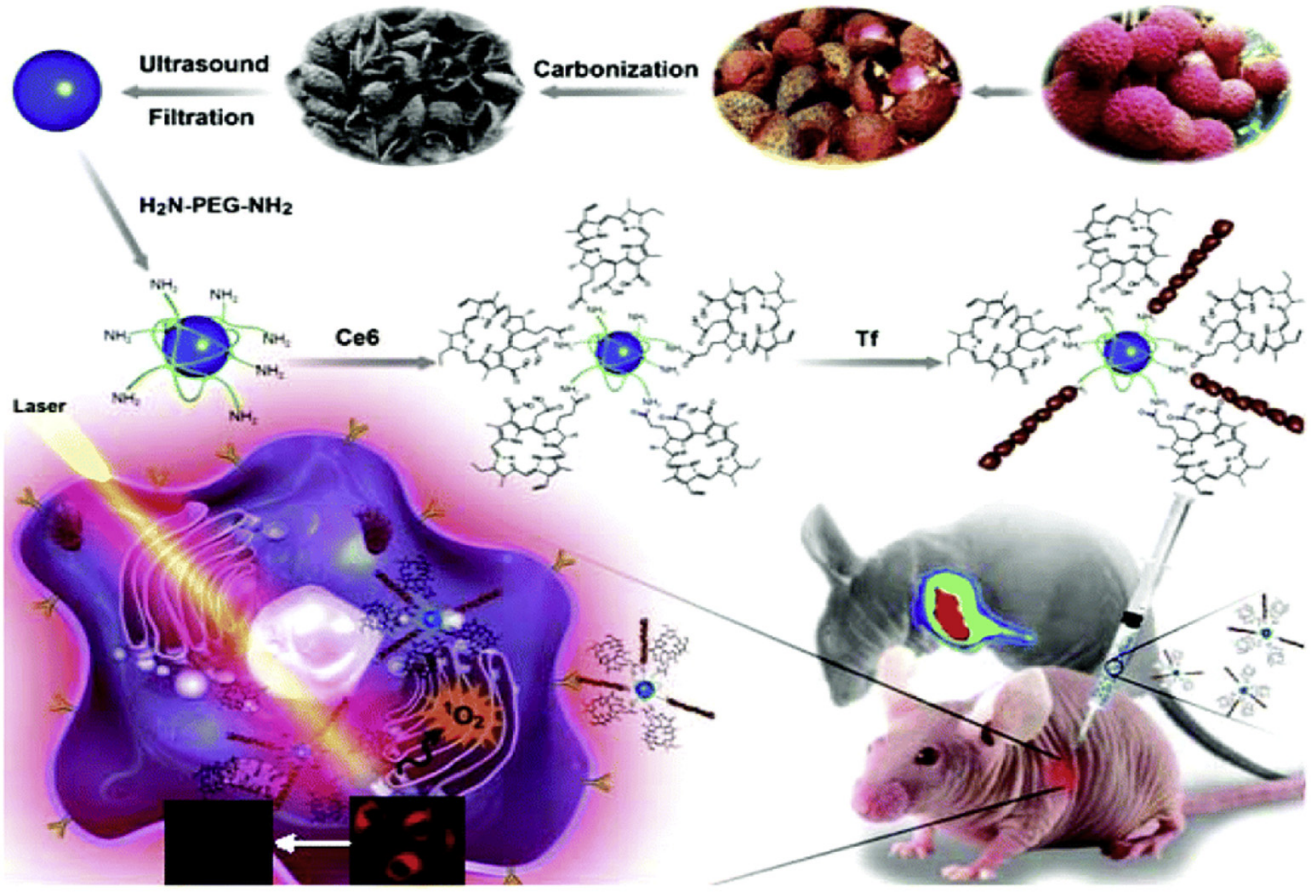
A schematic representation of the fabrication and application of natural biomass carbon dots (NBCDs) and NBCD-PEG-Ce6-Tf [217].

Nanocarbons, including fullerenes, CNTs, Gph, carbon dots (CDots), nanodiamonds and carbon nanohorns, are significant types of nanostructures attracting tremendous attention in the past decades (Fig. 13A). Owing to their highly enriched distinctive physical and chemical characteristics, nanocarbons have been widely applied in the field of biomedicine, such as PDT and PTT (Section 1.2.2.4) [200,201].

Fullerene discovered in 1985 is one of the typical sp^2^-carbon nanomaterials, which was composed of 60 or more carbon atoms arranged in a soccer ball–like structure. In such nanocarbons, the enriched aromatic rings induce broader π-conjugation of molecular orbitals, resulting in the strong absorbance of visible light [202,203]. Besides, fullerenes are also capable of generating ROS upon light irradiation, showing the potential as PS for PDT [204,205]. However, the major limitation of fullerenes applied for PDT is that their excitation has to be achieved by light with short wavelengths and high energy, undesirable for improving tissue penetration depth. To address this issue, Antoku at al. [206] encapsulated light-harvesting antenna molecules and fullerene derivatives into the lipid membrane. Through changing the location of the C60 derivative in the liposomal dyad system, an efficient photoenergy transfer from antenna molecules to the fullerene derivative occurred, leading to the improved photodynamic activity.

As another important type of nanocarbons, CNTs could also be used as PS for PDT [207,208]. In 2009, Naveen et al. observed that SWCNTs modified with -COOH and chitosan can generate ^1^O_2_ after nonlinear excitation [207]. They also found that several parameters including surface characters and content of residual iron catalyst would influence the ROS production. Based on their results, non-functionalized SWCNTs performed the better photodynamic activity than the functionalized ones. However, the surface modification of SWCNTs is essential when such nanostructures to be used *in vivo*, and it might not be that pragmatic to use the inherent photoinduced ^1^O_2_ production ability of SWCNT for PDT. Besides, some other researchers even observed that SWCNTs could quench ^1^O_2_ generation of PSs which were adhered on the surface of nanotubes. By use of unique property of SWCNTs, Zhi et al. [208] developed an interesting nanoplatform to regulate the ^1^O_2_ generation of PS-attached SWCNTs. PSs were conjugated to the end domain of DNA aptamer that winded onto the SWCNT surface. In non-targeted tissues, this system stays in inactive status, and SWCNTs suppress the ^1^O_2_ generation. However, upon treating the SWCNTs with target thrombin, the DNA aptamer could be detached from the SWCNT surface, leading to the recovery of ^1^O_2_ generation ability.

Besides, Gph is another typical kind of nanocarbons, in which every carbon atom is bonded to three neighboring carbon atoms by covalent bonds in a honeycomb-like ​pattern. In such nanocarbons, the unhybridized orbitals of carbon atoms are perpendicularly oriented to their planar structure and interact with one another to form a large system that gives its aromatic character [211,212]. The unique properties of Gph lead them to be used for potential PDT applications [[213], [214], [215]]. Dong et al. [213] first ​reported the PDT by using Gph-based NPs, in which ZnPc was embedded on the surface of PEGylated Gph (nGO-PEG) through π-π stacking and hydrophobic interactions. Based on their study, the resultant nGO-PEG-ZnPC revealed obvious photocytotoxicity against MCF-7 ​cells under Xe light irradiation. More recently, Chen et al. [216] reported the large-scale synthesis of crystalline gadolinium–encapsulated Gph carbon NPs (Gd@GCNs) showing a largely enhanced ^1^O_2_ quantum yield when applied for PDT. The resultant Gd@GCNs cannot only be used as a dual-modal contrast agent for both ﬂuorescence imaging and MRI. In particular, Gd@GCNs efficiently produce ^1^O_2_ under photoirradiation, suggesting their possibility for further clinical translation.

Recently, CDots ​with ultrasmall sizes are also used as novel nanocarbons for the application of phototheranostic nanomedicine [[217], [218], [219], [220]]. Huang et al. [219] designed Ce6-conjugated CDots for fluorescence imaging–guided cancer PDT. In such case, CDots were used for enhancing PDT efficacy through two distinct excitation pathways, which are FRET effect between CDots and Ce6 ​and direct activation of Ce6. It was concluded that this novel nanoplatform promoted the PDT efficacy against gastric cancer. In another work, Xue et al. [217] synthesized novel natural biomass carbon dots (NBCDs) using the exocarp of lychee ​and further encapsulated transferrin and Ce6 onto their surface to form the NIR fluorescence imaging nanoprobe (Fig. 13). The final NBCD-PEG-Ce6-Tf nanoprobes could emit NIR fluorescence and present desirable biosafety. After intratumoral injection of NBCDs, the Ce6 on their surface can produce ^1^O_2_ through photodynamic activation upon laser irradiation, leading to the cell death and inhibited tumor growth in PDT-treated mice.

#### 2.5.6 Upconversion NPs

Upconversion nanoparticles (UCNPs), also known as lanthanide-doped NPs, could emit higher energy photons when irradiated by light with lower energy. Compared with conventional downconversion fluorophores, the UCNPs display the strengths, such as enhanced tissue penetration, higher photochemical stability, and photochemical stability, weak autofluorescence interference, facilitating them to apply in biomedical imaging and cancer PDT [221]. Many research groups have developed PDT strategies based on UCNP-PS nanocomplexes [[222], [223], [224]]. The first research about *in vivo* UCNP-involved PDT was conducted by Wang et al. [222]. They encapsulated Ce6 into PEGylated polymer–coated UCNPs and intratumorally injected the resultant UCNP–Ce6 into 4T1 tumors bearing mice. As per their results, the remarkable therapeutic efficiency was found after PDT treatment mediated by a 980 ​nm light. Moreover, the injected UCNPs could be continuously cleared out from normal tissues 2 months after injection, demonstrating the well biosafety of such UCNPs.

**Fig. 14:**
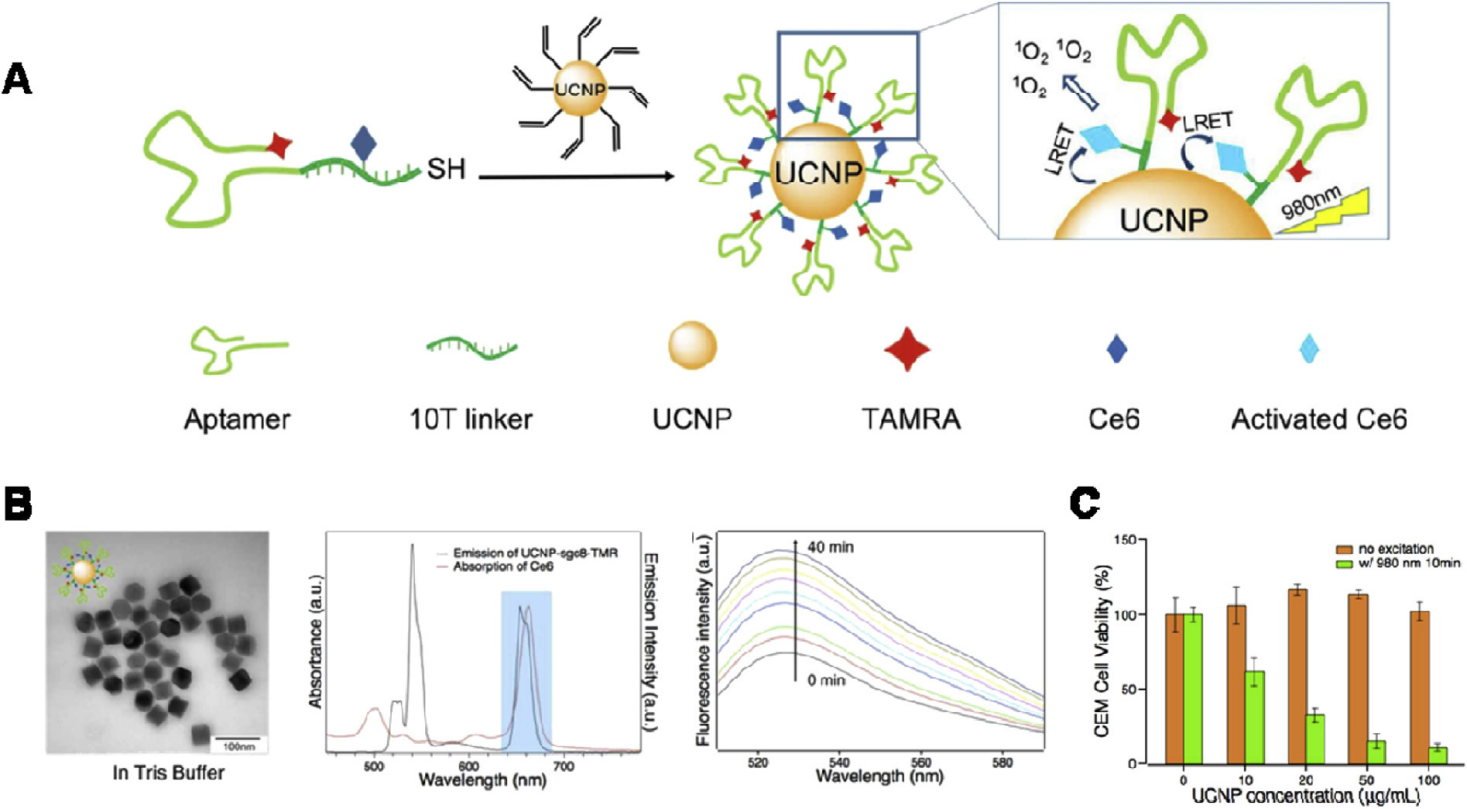
(A) Schematic illustration of the preparation of UCNP–Ce6-aptamer and its ROS generation principle. (B) TEM images of UCNP with DNA conjugation, the emission spectrum of UCNP (black line) and UV-vis absorption spectrum of Ce6 (red line), and monitoring ^1^O_2_ generation of UCNP-Ce6-sgc8 upon 980 ​nm laser irradiation, in sequence from left to right. (C) PDT efficacy of CEM cells treated with UCNP-Ce6-sgc8 [227].

Besides, UCNP-based PDT could also be realized through systemic administration [225]. Sisi et al. [226] developed tumor-homing UCNP-PS nanosystems with a high content of ZnPc and used the obtained nanostructures for deep-tissue PDT of cancer cells. In their work, folate-functionalized chitosan(FASOC) was synthesized for the surface modification of UCNPs as well as ZnPc binding, although facilitating the FRET effect between UCNPs (donor) to ZnPc (acceptor). When compared with PDT using red light as a light source, such an NIR light–triggered PDT displayed higher tumor suppression efficacy. More recently, Hou et al. [227] used multifunctional aptamers as targeting ligands for UCNPs surface modification (Fig. 14). The resultant nanodrugs presented high tumor-homing ability and desirable cytotoxicity even at relatively low drug concentrations, which was accounted for the following reasons: (1) the close proximity of UCNP and PS promoted the high FRET efficiency, (2) an open NP surface enabled the fast diffusion of the generated ^1^O_2_, (3) covalent drug conjugation strategy minimized the PS premature release, and (4) effective cell internalization of nanodrugs was achieved because of the targeting effect of aptamers.

### 2.6 Others

#### 2.6.1 Porphysomes

**Fig. 15:**
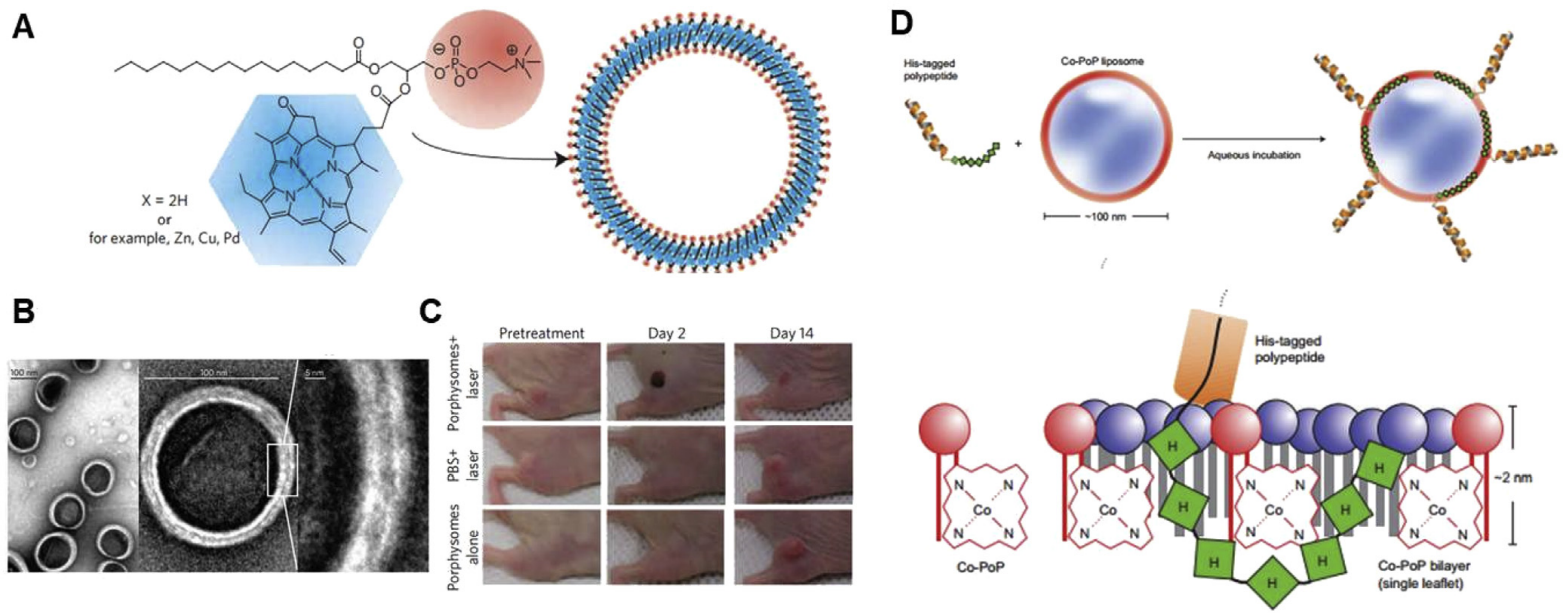
(A) Chemical structure of pyropheophorbide-lipid used to form porphysomes. (B) TEM images of porphysomes. (C) Photographs showing therapeutic response to PTT by porphysomes [97]. (D) Polyhistidine-tagged polypeptides bind and functionalize Coporphysomes [232]. TEM, transmission electron microscopy; PTT, photothermal therapy.

In 2011, Lovell et al. [97] first ​reported a novel porphyrin-lipid nanostructures named porphysomes, since then a series of systematical studies about this nanoplatform have been conducted to reveal their superiorities. As shown in Fig. 15A, porphysomes are self-assembled from the porphyrin-lipid (pyro-lipid), which generated by an acylation reaction between lysophosphatidylcholine and pyropheophorbide, a chlorophyll-derived porphyrin analog. The firmly packed pyro-lipid dyes embedded within the porphysome bilayer lead to an impressively high molecular absorption coefficient (∼10^9^ ​M^−1^) and excited state quenching efficiency (>99%). Owing to this aggregation state, the photodynamic activity of porphyrin quenched significantly and hence, generated only a small amount ROS upon light irradiation. However, once the porphysomes encounter some special circumstances, such as a cellular compartment of a tumor, their PDT capacity could restore to induce cell death [97,228,229].

Besides, the researchers also discovered that the quenched property of porphysomes in their intact state enables them to use vibrational relaxation to dissipate the absorbed energy, resulting in heat generation. The photothermal conversion abilities of porphysomes were investigated by Lovell [97] and Jin [230]. KB tumor-bearing mice were treated with porphysomes followed by the light irradiation (671 ​nm, 1.9 ​W ​cm^−2^, 60 ​s) at 24 ​h after tail vein injection. Based on the results, an enhancement in tumor temperature of 30 ​°C was observed and no tumor recurrence during the experiment (Fig. 15C). The switching from photodynamic action to PTT of porphyrin molecules in porphysomes is beneficial to treat hypoxic tumors when compared with PDT, which is an oxygen-dependent therapeutic modality [231]. Based on Zheng's research, which directly compared the PDT and PTT efficacy of porphysomes in mice bearing hypoxic tumors, PTT could induce the ablation of tumors without regrowth. However, as for mice undergoing PDT, there was no survivor on day 20. These results indicate that porphysomes-mediated PTT can address the issues along with hypoxic tumor treatment to some extent.

Moreover, porphysomes exhibit a long blood clearance half-life, which was investigated by using the Cu^64^ radiolabelled porphysomes [233,234]. Through recording blood radioactivity at predetermined time points after injection of porphysomes, the blood clearance half-life of porphysomes was 1.23 ​h (first phase) and 11.1 ​h (second phase). Accordingly, mice treated with porphysomes at dose of 1000 ​mg/kg showed negligible abnormal behaviors and weight loss. Besides, the results from other tests, such as liver function tests were all found in their normal range [97].

Modification of polypeptides onto the surface of porphysomes could further render them more functions. However, methods to attach polypeptides to lipid bilayers are often ineffective. Considering the advantages of polyhistidine tag (his-tag) on binding to immobilized metals, Shao et al. [232] reported an interesting strategy in which the his-tag was conjugated to the terminal of a polypeptide. Owing to the presence of cobalt chelated in the porphyrin-phospholipid conjugates, the his-tag proteins and peptides could be captured in the lipid bilayers. Notably, the binding induced a Co(II) to Co(III) transition in the hydrophobic domain, leading to an essentially irreversible attachment even in a complex physiological environment (Fig. 15D). In summary, porphysomes showed potential as promising nanophototherapeutics for further clinical application.

#### 2.6.2 Cargo-free nanophototherapeutics

Cargo-free nanomedicine is prepared through self-assembly of therapeutic agents in the absence of external excipients. The new developed nanomedicine not only takes advantage of nanomedicine aforementioned but also shows some other superiorities, such as high drug-loading efficiency, desirable biosafety, deep tumor penetration, large-scale fabrication and so on, which have been comprehensively discussed in a previous review [235]. Owing to the advantages aforementioned , cargo-free nanomedicine has been considered a promising strategy for future clinical applications [236].

Owing to the features of cargo-free nanomedicine, researchers have also attempted to develop cargo-free nanophototherapeutics for efficient phototherapy. Li et al. [237] constructed NPs NanoPcTBs through self-assembling the Pcs monomers Pc-4TEG-B. The NanoPcTBs display inherently unique photothermal and PA ability. Fluorescence and ^1^O_2_ production could be induced via a protein-induced mechanism and partial disassembly mechanism, creating opportunities for low autofluorescence interference imaging and targeted PDT. As for another interesting work, Wang et al. [238] prepared ZnPc NPs by using other ZnPc monomers. The as-prepared ZnPc NPs were found to be stable and biocompatible, which also display ​remarkable PDT efficacy and high photothermal conversion efficiency (as high as 31.3%) upon NIR light irradiation. The simple synthetic procedure and prominent therapeutic effect from PDT/PTT make the as-formed ZnPc NPs a potential cargo-free nanophototherapeutic for cancer diagnosis and treatment.

**Fig. 16:**
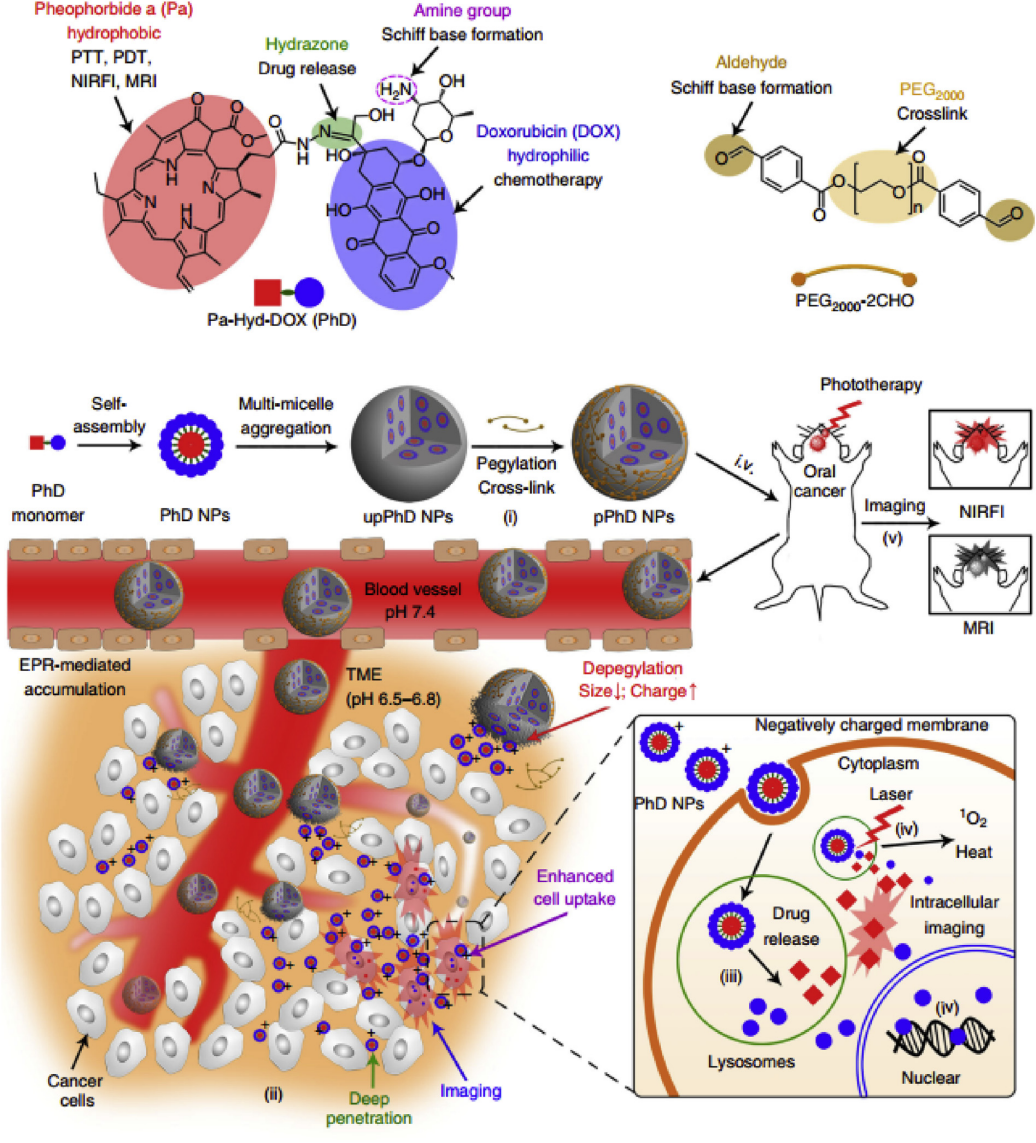
Schematic illustration of the functionalities of the PhD monomer and construction of the pPhD NPs, and the mechanism behind overcoming biological barriers via TME sensitivity, dePEGylation/cross-linking, transformability, improved penetrations, enhanced cellular uptake, controlled drug release for multimodal imaging, and trimodal PDT/PDT/chemotherapy [240]. TME, tumor microenvironment; NPs, nanoparticles; PDT,photodynamic therapy; PTT, photothermal therapy.

Instead of using photoagents as a building block to construct NPs, transferring them into prodrug is another promising strategy for preparing the efficient cargo-free nanophototherapeutic. Guo et al. [239] developed a bioreductive prodrug consisting of PS and angiogenic vessel–targeting peptide. Through self-assembly of the prodrug, the angiogenesis vessel–targeting nanoparticle (AVT-NP) could be obtained while loading hypoxia-activated drug TPZ. During the PDT process, tumor hypoxia could be induced, resulting in the upregulation of vascular endothelial growth factor ​at the tumor tissues. With targeting ability, the AVT-NP can extensively localize at the tumor sites because of the accelerated angiogenesis. The more NPs accumulated at tumor tissue, the better performance of PDT can be realized, leading to more serious hypoxia and enhanced angiogenesis. Hence, the prodrug-loaded AVT-NP played the function of positive feedback amplifier during chemo/PDT and achieved impressive antitumor efficacy. More recently, Xue et al. [240] reported a dual size/charge transformable, Trojan-Horse NP, named pPhD NPs, for dual-modal imaging–guided trimodal therapy. As shown in Fig. 16, an amphiphilic molecule (PhD monomer) was first ​synthesized by conjugating PS Pheophorbide a (Pa) to DOX through the acid-liable hydrazone linker. Subsequently, PhD was used for self-assembly of micelles PhD NPs and further micelle aggregation of upPhD NPs. Then, dual-aldehyde–terminated PEG (PEG-2CHO) is reacted with the primary amine groups on the surface of upPhD NPs whereas cross-linking the NPs through the formation of pH-sensitive Schiff base bonds to afford final pPhD NPs. In acidic TME, the dePEGylation of pPhD NPs dramatically promote the tumor penetration and cellular internalization. Once located into the acidic lysosomes in the cancer cells, the cleavage of hydrazone bond occurred and both PS/DOX could be released to amplify their therapeutic efficacy. Accordingly, the synergistic PDT/PTT/chemotherapy achieved a complete eradiation of both subcutaneous and orthotopic oral tumors, indicating a powerful, efficient, and versatile nanoplatform was developed and waiting for ​further clinical detection.

#### 2.6.3 Peptide- and protein-based nanophototherapeutics

Peptides and proteins are promising building materials for nanostructures, presenting inherent well-ordered configuration [241]. Usually, the noncovalent interactions, including π-π interaction, Van der Waal forces, ionic attraction, hydrophobic effect, and hydrogen bonding, facilitate the self-assembly of peptides and proteins, which could be elaborately controlled by altering the sequences of amino acids and controlling the environmental conditions [242]. Besides, these self-assembled NPs showed diverse improvements when compared with their building blocks [243]. Therefore, the establishment of peptide- and protein-based nanophototherapeutics are receiving enormous interest recently to fulfill the specific requirements of phototherapy.

**Fig. 17:**
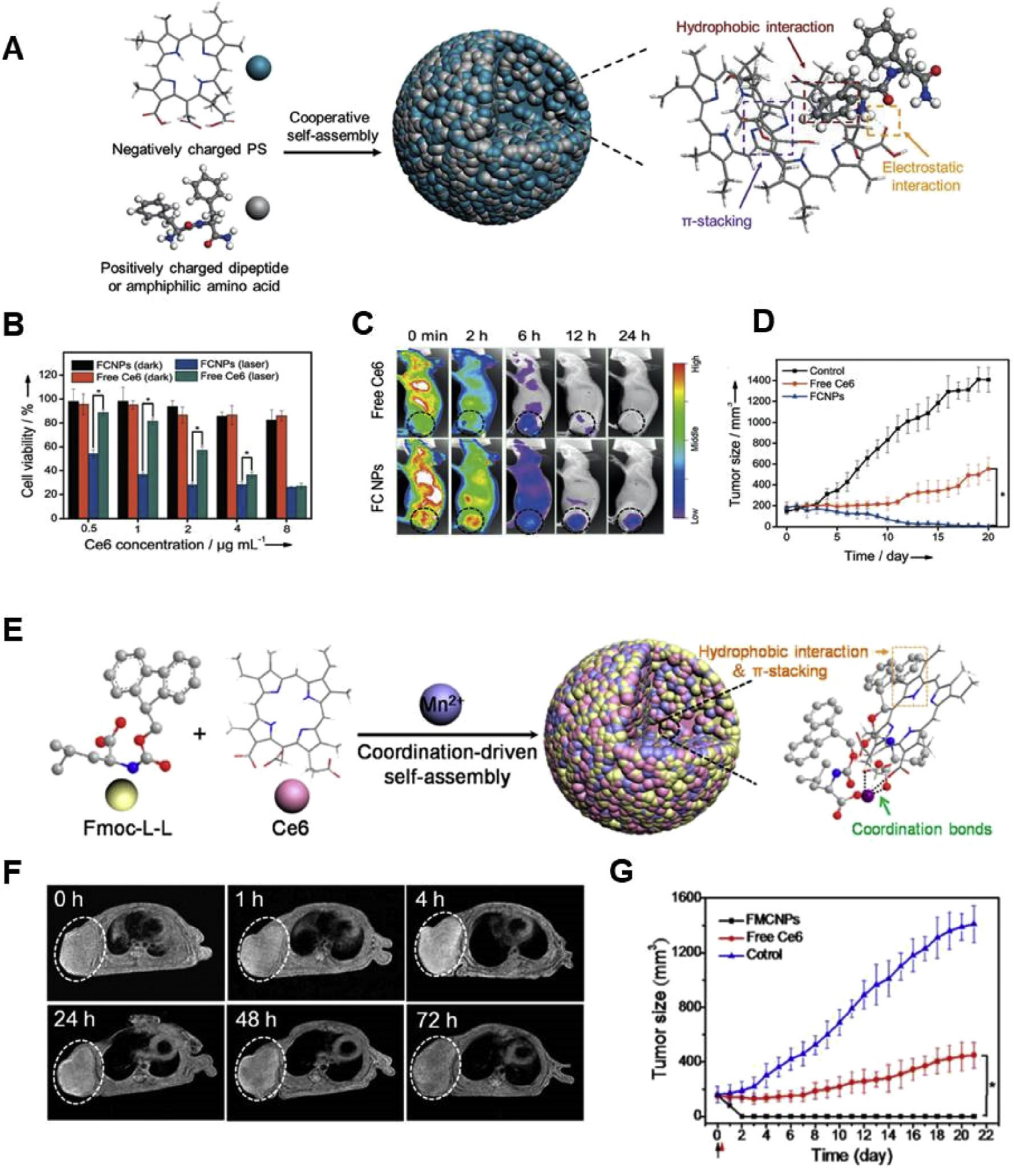
(A) Formation of phototheranostic nanoassemblies FCNPs via amphiphilic dipeptide– or amino acid–tuned self-assembly. (B) Photocytotoxicity of FCNPs in light or dark with free Ce6 as control. (C) Biodistribution of FCNPs in MCF7 tumor–bearing nude mice. (D) Measured tumor growth for 20 days after treatment [244]. (E) The fabrication process of FMCNPs via coordination-driven self-assembly. (F) T_1_-weighted magnetic resonance images of nude mice bearing MCF7 breast cancer xenografts after intravenous injection of FMCNPs. (G) Measured tumor growth of mice bearing MCF7 breast cancer xenografts after intravenous injection of FMCNPs and free Ce6 [245].

Recently, Liu et al. [244] revealed that short peptide could self-assemble with PSs to form efficient nanophotosensitizers [[244], [245], [246], [247]]. They demonstrated that cationic amphiphilic peptides with aromatic groups, such as cationic diphenylalanine (CDP) and an amino acid derivative (Fmoc-L-Lys), can promote the self-assembly of negative Ce6 into NPs through different interactions (Fig. 17A–D). The morphology of resultant Fmoc-L-Lys/Ce6 (FCNPs) and CDP/Ce6 (CCNPs), with sizes around 200 ​nm and 100 ​nm, can be well adjusted by altering the peptides/PSs molar ratio. Importantly, these NPs showed stimuli-responsive properties, leading to the selective release of PSs in a well-controlled behavior. *In vivo* PDT results also revealed that both NPs could induce significant tumor ablation with negligible side-effect, indicating their high biocompatibility. As an extension of previous work, Zhang et al. [245] ​further fabricate a supramolecular nanoplatform (FMCNPs) by self-assembly of the amphiphilic amino acid Fmoc-modified leucine (Fmoc-_L_-L)-modulated and Ce6 in the presence of a MRI contrast agent (ionic manganese, Mn^2+^) (Fig. 17E). Based on their research, coordination drove the coassembly of Fmoc-L-L and Mn^2+^ to form nanoplatforms to further encapsulate Ce6. Besides, the obtained NPs were found to be unstable under the environment with a high level of GSH via the competitive coordination of GSH with Mn^2+^. The antitumor process can be monitored and evaluated by MRI through the long-term intracellular biochelation of Mn^2+^ (Fig. 17F) and the PDT against MCF7 breast cancer was also successful accordingly (Fig. 17G).

Protein molecules could also be used as carrier materials for preparing phototheranostic nanomedicine. For example, human serum albumin (HSA) in the human body is the natural building material ​for delivering anticancer drugs and photoagents [[248], [249], [250]]. Chen et al. [250] tuned the photophysical properties of IR825 by encapsulating this dye into HSA. The HAS-IR825 complexes were acquired by combing HSA with IR825 at an optimized molar ratio (1:1). Interestingly, the HAS-IR825 complexes also displayed the wavelength-dependent ﬂuorescence properties, which was good for selective ﬂuorescence imaging and PTT. Upon 600 ​nm excitation, *in vivo* ﬂuorescence signals could be captured to evaluate the biodistribution of complexes. However, efficient tumor ablation occurred when changing the light source to laser with wavelength at 800 ​nm.

## 3 Particular applications in cancer treatment

### 3.1 Medical imaging

With the advancement of personalized cancer treatments, it is quite necessary to develop more superior imaging modalities for cancer diagnosis concurrently. Besides, efficient integration of diagnosis and treatment now seems to be the trend for clinical application. Phototheranostic nanomedicine is particularly suitable for filling the vacancies. Various contrast agents have been incorporated into the NPs to increase medical imaging quality. In presence of sophisticated nanoplatforms, the imaging modes, including fluorescence imaging, MRI, PA imaging, computed tomography, and positron emission tomography ​and so on, were successfully realized for guiding the cancer therapy. The representative examples have been listed in Table 2 for a better readership. Fluorescence imaging, MRI, and especially PA imaging are discussed in the following parts.

**Table 2:** Selected examples of phototherapeutic nanoplatforms for cancer diagnosis and treatment.

Reference.	Type of nanoparticles	Therapeutic modalities	Imaging modalities	PSs	PTT/PA agents
[115]	Polymeric micelles	PTT	Fluorescence		IR825
[117]	Polymeric micelles	PDT		Ce6	
[118]	Polymeric micelles	PDT		ZnPc	
[119]	Polymeric micelles	PDT+Chemo		ZnPc	
[120]	Polymeric micelles	PDT+Chemo		Hp	
[121]	Polymeric micelles	PDT		Ce6	
[123]	Polymeric micelles	PDT+Chemo		Ce6	
[96]	Polymeric nanoparticles	PTT			PCCP
[130]	Polymeric nanoparticles	PTT+Chemo			ICG
[134]	Polymeric nanoparticles	PTT			Polypyrrole
[135]	Polymeric nanoparticles	PTT			SPNs
[138]	Polymeric nanoparticles	PDT		Ce6	
[141]	Liposomes	PDT		Dimeric BODIPY	
[150]	Liposomes	PDT+PTT+Chemo		ICG	ICG
[159]	Nanogels	PDT		HPPH	
[90]	Dendrimers	PDT+PTT		SiNc	SiNc
[170]	Dendrimers	PDT		RB and PpIX	
[182]	Silica NPs	PDT		ZnPc	
[209]	Nanocarbons	PDT		Ce6	
[210]	Nanocarbons	PDT		DPP	
[226]	Upconversion NPs	PDT		ZnPc	
[229]	Porphysomes	PDT		porphyrin	
[250]	Peptide- and protein-based nanophototherapeutics	PTT			IR825
[194]	Magnetic nanoparticles	PDT+Hyperthermia	MRI	Pheophorbide-a	
[195]	Magnetic nanoparticles	PDT		Ce6	
[205]	Nanocarbons	PDT		Fullerene	
[136]	Polymeric nanoparticles	PTT	PA	Conjugated polymer	
[231]	Porphysomes	PDT+PTT		porphyrin	porphyrin
[270]	Bacterial outer membrane vesicles (OMVs)	PTT			Melanin
[271]	Metallic nanoparticles	PTT+Chemo			Gold nanorods
[122]	Polymeric micelles	PTT+Chemo	Fluorescence+PA		IR780
[129]	Polymeric nanoparticles	PTT+Chemo			ICG
[137]	Polymeric nanoparticles	PDT+PTT		(SPNs)	SPNs
[237]	Cargo-free nanophototherapeutics	PDT+PTT		ZnPc	ZnPc
[248]	Peptide- and protein-based nanophototherapeutics	PTT			CySCOOH
[216]	Nanocarbons	PDT	Fluorescence+MRI	Graphene	
[241]	Cargo-free nanophototherapeutics	PDT+PTT+Chemo		Pheophorbide-a	Pheophorbide-a
[245]	Peptide- and protein-based nanophototherapeutics	PDT		Ce6	
[140]	Liposomes	PTT	PA+MRI		Melanin
[267]	Bovine serum albumin nanoparticles	PTT			CuS
[124]	Polymeric micelles	PDT+PTT	Fluorescence ​+PA+MRI	TCPP	IR825
[142]	Liposomes	PDT+PTT	Fluorescence ​+PA+CT	IR780	IR780
[143]	Liposomes	PDT+PTT		ICG	ICG

Abbreviations: HPPH, 2-devinyl-2-(1-hexyloxyethyl) pyropheophorbide; Hp, Hematoporphyrin; PCCP, porphyrin-containing conjugated polymer; SNPs, semiconducting polymer nanoparticle; SiNc, silicon naphthalocyanine; RB, rose bengal; PpIX, Protoporphyrin IX; DPP, Diketopyrrolopyrrole; TCPP, 5,10,15,20-tetrakis (4-carboxyphenyl) porphyrin; PDT, photodynamic therapy; ICG, indocyanine green; PTT, photothermal therapy; Chemo, chemotherapy; IR, ionizing radiation.

Fluorescence imaging, as the most cost-efficient imaging mode, has improved fast in recent decades by using the excitation characters of fluorophores. Because many phototherapeutics display the fluorescent property, the NPs encapsulating these components (e.g. NIR dyes, SPNs, QDs) could serve as fluorescent contrast agents for real-time cancer fluorescence imaging with increased spatial resolution [251,252]. Noteworthily, it is a fact that NIR-II fluorescence imaging presents higher spatial and temporal resolution than the NIR-I fluorescence imaging because of the decreased absorption and scattering by tissues and preventable autofluorescence in corpora. Hence, researchers attempted to introduce the NIR-II fluorescent dyes into phototherapeutic NPs to obtain the anticipated NIR-II fluorescence performance [126].

MRI depends on the ability of the magnetic dipoles of water protons to align under the action of a strong magnetic field. The relative physical principles, image acquisition, and processing have been discussed in the previous works [253,254]. Among various imaging modalities, MRI is known for its advantage in good contrast in soft tissue and ability to give the readers more details related to tissue function, structure, blood perfusion, and so on. Superparamagnetic iron oxide nanoparticles (SPION), approved by the FDA, can serve as MRI contrast agents for cancer diagnosis and therapy by providing a strong T_2_ contrast effect becasue of their magnetic inhomogeneity [255]. Through the incorporation of SPION into phototherapeutic nanosystems, the MRI-guided phototherapy could be realized with examples listed in Table 2. Besides, metal ion–chelated MRI contrast agents have also drawn much attention. Accordingly, gadolinium(III)-based contrast agents (GBCAs)are used in around 40% of all MRI examinations, representing about 40 million administrations of GBCAs worldwide. Chelating Gd (III) into phototherapeutics or carrier materials gives phototherapeutic nanosystems their function for MRI [256].

PA imaging is a new fashioned hybrid imaging technique by incorporating optical imaging with ultrasound, expanding the imaging depth to several centimeters [257]. In the process of PA imaging, the endogenous or exogenously administered light absorber in the desired location is excited by the short-pulsed laser firstly and partially converts the energy into heat via vibrational relaxation. Subsequently, the sound waves are generated through thermoelastic expansion, which is then collected by an ultrasound transducer to perform the three-dimensional reconstruction. Finally, the PA images can be obtained in accordance with arrival times of sound waves to a transducer. As compared with photons, phonons are hardly scattered in biological tissues. In addition, the scattered photons during PA imaging could also promote the production of PA waves [258]. Therefore, PA imaging possesses the combined advantages of acoustic imaging of high spatial resolution and optical imaging of high contrast. So far, PA imaging is undergoing clinical translation, such as early diagnosis of prostate and breast cancer [259], detection of tumor metastases [260], endoscopic gastrointestinal imaging, and treatment monitoring [261]. Although PA imaging is capable of performing diagnosis in the absence of contrast agents, such as endogenous Hb and melanin that can generate PA signals by themselves, most of the biological tissues are inherently low contrast because of relatively weak absorption in the NIR range. To further increase its use in the diagnosis of disease, external PA imaging agents with high absorption coefficients and tumor selectivity have been developed, particularly NPs absorbing light in BWs [262].

**Fig. 18:**
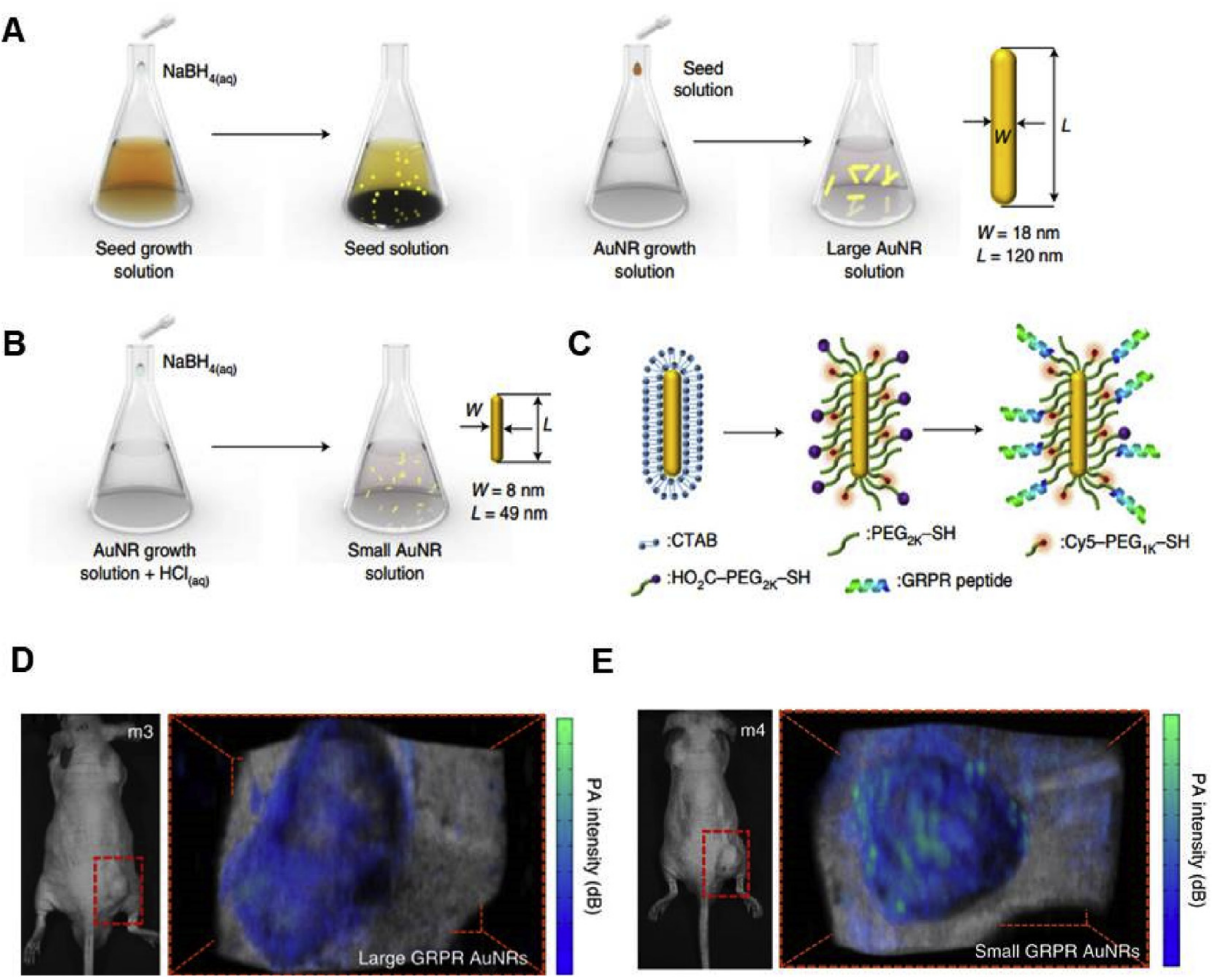
(A) Illustration of traditional seed-mediated growth procedures for producing large GNRs. (B) Illustration of seedless growth procedures for producing small GNRs. (C) Illustration of surface functionalization of the GNRs for targeting GRPR on prostate cancer cells. (D and E) Photographs (left) and PA imaging (right) of tumor-bearing mice with targeted large (D) and small (E) GNRs [272]. GNRs, gold nanorods; PA, photoacoustic; GRPR, gastrin-releasing peptide receptor.

Owing to the fact that PA agents play the function of efficient light-to-heat conversion, which is also involved in PTT, most of the PTT agents (Section 1.2.2) are actually promising PA imaging agents, such as gold nanoclusters [263], CNTs [264], reduced Gph oxide [265], UCNPs [266], QDs [267], small-molecule dyes [268], SPNs [269], melanin [270], and so on. For example, GNRs exhibit excellent and tunable optical properties, making them good candidates for PA imaging [271,272]. Very recently, Chen et al. [272] reported a kind of miniaturized GNRs which could enhance the PA contrast as compared with the regular-sized GNRs. As shown in Fig. 18A–C, seedless approach was used to synthesize the miniature GNRs with absorption in the NIR-II and much smaller than regular ones in an alike aspect ratio (length/width). Upon illumination by nanosecond pulsed laser, miniaturized nanorods are much more stable and could generate a strong PA signal than the corresponding regular GNRs. In a tumor-bearing mice model, the miniaturized GNRs presented a 30% enhancement in the efficiency of agent delivery and generated much stronger PA signals (Fig. 18 D and E). In accordance with the conclusion derived from the theoretical and numerical analysis by authors, the PA signal is not only relevant to the absorption of GNRs but also to their surface-to-volume ratio.

Organic PA agents especially small molecule dyes have been widely studied because of their superiorities, including their versatility of modification, good biocompatibility, and relatively low cost, making them potential PA agents [273,274]. However, their poor photostability compromises their further application. Most recently, SPNs (Section 2.1.2), with high photostability and well-controlled optical properties have emerged as an alternative kind of PA agent, attracting the attention of researchers worldwide [269,275]. Recently, Jiang et al. [269] also focused on developing the SNPs-based NIR-II absorbing agents, as PA agents, to reduce light scattering and minimize tissue absorption when performing PA imaging. They first ​synthesized a series of the π-conjugated SPs and then mixed with hydrolyzable amphiphilic polymer PLGA-PEG to obtain the firstly reported NIR-II PA agents. Accordingly, these PA agents presented high photothermal conversion capacity and legible PA images at 1064 ​nm, making them suitable for PA detection on both subcutaneous tumor and deeper brain vasculature at a low dosage of PA agents.

**Fig. 19:**
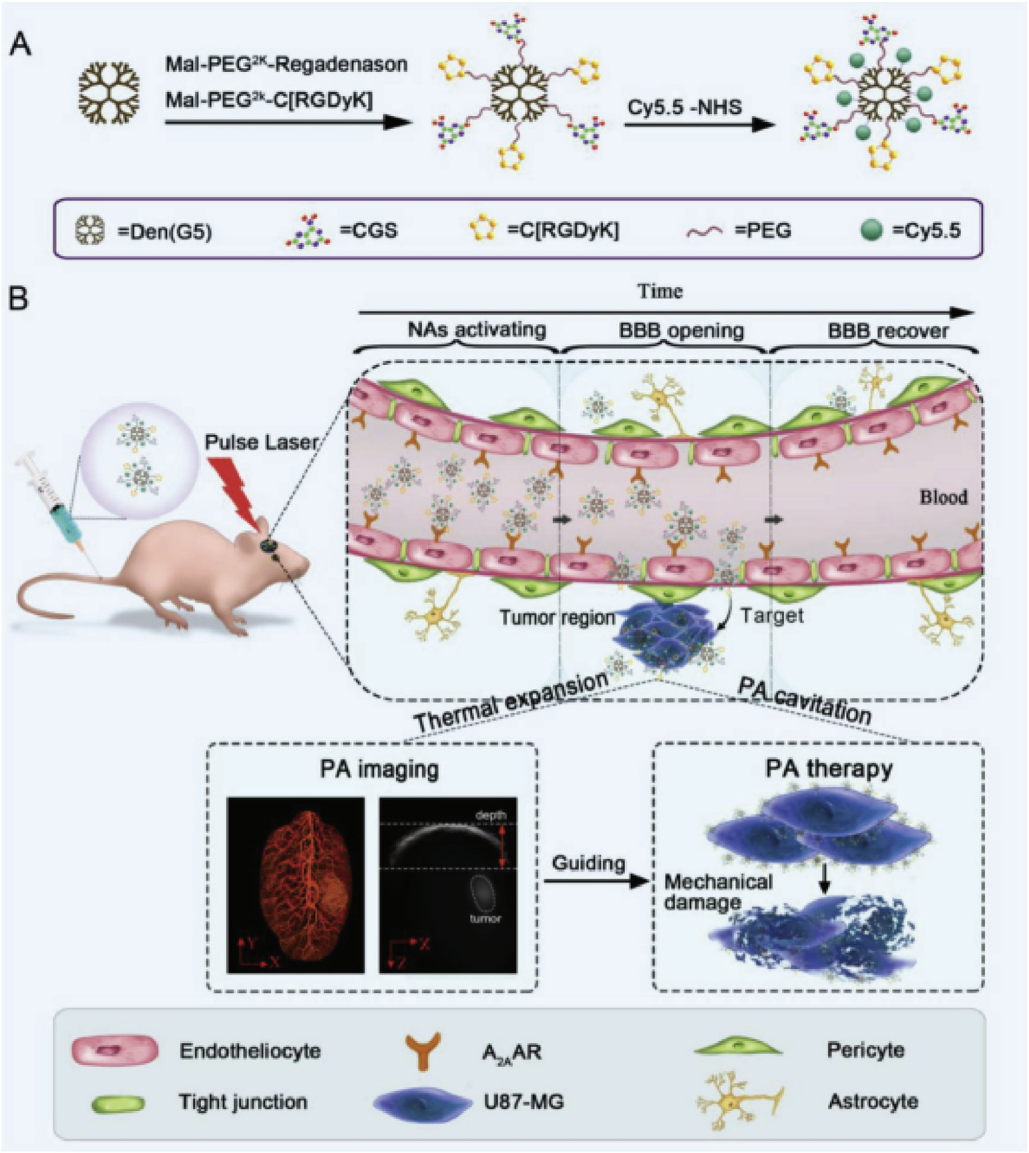
(A) Schematic summary of the synthesis of the Den-RGD/CGS/Cy5.5 nanoparticle. (B) Schematic illustration of photoacoustic (PA) precision glioblastoma therapy with Den-RGD/CGS/Cy5.5 [276].

Notably, PA cavitation could also be used to achieve mechanical damage on target tissues, leading to a therapeutic outcome. Recently, Liu et al. [276] prepared a dendrimer-based NP with high red absorbance and excellent penetration of the blood-brain barrier (BBB) in tumor tissues to overcome the difficulties when treating glioblastoma (Fig. 19). The presence of 4-[2-[[6-Amino-9-(*N*-ethyl-â-d-ribofuranuronamidosyl)-9*H*-purin-2-yl]-amino]ethyl]benzenepropanoic acid hydrochloride (CGS) on the surface of NPs could timely activate adenosine receptor on the BBB to facilitate self-accumulation in the tumor. Besides, the NP converted pulsed laser energy into a shockwave via PA, leading to a precision antitumor effect. More importantly, the NP-mediated PA process can also assist researchers to visualize the tumor depth, size, and vascular morphology.

### 3.2 Phototherapy against cancer stem cells

On a cellular level, the cause of cancer is a genetic mutation. The errors in DNA instructions endow cells to evade normal cell cycle functions particularly cell cycle arrest. Even so, it has been reported that malignant tumors can be caused by a subpopulation of cells (1%) in tumor tissues, called cancer stem cells (CSCs), which originate from normal stem cells (SCs) that underwent genetic and epigenetic changes and by dedifferentiation from somatic tumor cells. It is also found that these CSCs, characterized by strong self-renewal ability and proliferation capacity, are responsible for tumorigenesis, tumor maintenance, tumor spread, and tumor relapse. In addition, CSCs have various capabilities allowing them to survive conventional cancer treatment including chemotherapy and radiotherapy [277]. Therefore, other new developed therapeutic modalities (e.g. phototherapy) have been an alternative approach for eliminating CSCs [278]. For example, Atkinson et al. [279] treated patient-derived xenografts by combining the ionizing radiation (IR) and PTT, and the results indicated that mild hyperthermia (42 ​°C) via AuroLase therapy can sensitize breast cancer stem cells (bCSCs) to IR, which may be due to PTT changing heat shock protein (HSP) expression of bCSC and impairing their ability to repair double-stranded breaks in DNA caused by IR. In addition, Paholak et al. [280] evaluated the ability of PTT via highly crystallized iron oxide NPs to inhibit bCSCs to inhibit metastasis after treatment of the primary tumor. Their results revealed that the potential of PTT mediated by nanoagent could enhance CSC destruction and improve long-term survival in patients with breast cancer ​and also provide a viable treatment option for those with currently incurable metastatic disease.

Moreover, to further enhance the inhibition efficiency against CSCs, phototherapy combined with other current clinical treatments was conducted and studied. Chang et al. [279] reported that photothermal-based nanoagents were used to enhance the sensitivity of CSCs to IR. In their work, mice with breast tumors were irradiated by a single dose of Gy first ​and laser subsequently to induce the temperature enhancement to 42 ​°C mediated by intravenous injection of GNSs. Based on their analysis, the combination of radiotherapy and PTT significantly reduced the percentage of CSCs than single radiotherapy. Furthermore, although PTT alone had no therapeutic effect on tumor growth and single radiotherapy suppressed the tumor growth insufficiently, the combined therapy led to obviously higher tumor growth inhibition. PTT could not only block the HSP90 ​and AKT pathway but also influence the TME and damage CSC alcove. The combination of heat and chemotherapeutics could result in synergistic therapeutic effects, a new interesting strategy was developed for killing CSCs, which was using thermoresponsive NPs to carry anticancer drugs. For example, Xu et al. [281] encapsulated CSCs inhibitor salinomycin (SA) in polydiallyldimethylammonium chloride (PDC)–conjugated GNRs (Au/SA@PDC) for combinational PTT/chemotherapy upon NIR laser irradiation. Accordingly, the combination of Au/SA@PDC plus NIR laser decreased the population of CSCs to zero, mainly because of the increased release of SA during the PTT process. Besides, GNRs-mediated PTT also resulted in the downregulation of ALDH^+^ cell subpopulation, ALDH1, and KLF4expression (SC markers), and mammosphere forming ability.

Aside from PTT, PDT also showed promising potential for inhibiting CSCs. An interesting work has been carried out by Usacheva et al. [282] by the incorporation of MB into alginate-Aerosol OT NPs to increase the ROS generation under hypoxic environment via type I mechanism (Section 1.1.1). As a consequence, PDT with MB NPs more effectively eliminated CSCs under either normoxia or hypoxia when compared with free MB. More recently, Wang et al. [283] developed a novel C60 fullerene-silica nanosystem coated by HA for targeting CD44 overexpressed bCSCs. Moreover, DOX and ICG were loaded in the NPs to achieve combined chemotherapy/PDT/PTT under NIR laser irradiation, finally effectively destructing the bCSCs with a negligible side-effect on normal tissues. They also found that the NPs are qualified candidates for augmenting cancer therapy by eliminating the CSCs.

### 3.3 Phototherapy-synergized cancer immunotherapy

Cancer immunotherapy, specifically anticancer vaccines and immune checkpoint blockade, has obtained more interest recently becasue of their potential clinical efficacy [[284], [285], [286], [287]]. However, there is still a long way to go before completely eradicating primary and distant tumors by using single cancer immunotherapy, mainly because of the challenges in identifying the high-efficient biomarkers as well as considerable patient individual differences. To further improve the therapeutic effect, the combination of multiple treatments to obtain a synergistically therapeutic outcome is a promising strategy [288], in which phototherapy-synergized cancer immunotherapy occupied an important position [289,290]. It has been reported that phototherapy could promote the antitumor immune response and activate the memory of immune cells during the process of treatment. Before understanding the specific research examples, understanding the process that PDT/PTT-assisted tumor immunotherapy, which could induce a cascade effect of immune cells in the TME, is very necessary [291]. PDT/PTT would induce a great diversity of antigens released from tumor lesions, which could enter into the blood circulation subsequently. However, it is worth noting that laser irradiation dosage also plays an important role in damaging immune system cells and surrounding tissues. For example, hyperthermia (>50 ​°C) induced by PTT can potentially cause inflammatory disease and heating damage of normal organs nearby; thus, it is better to perform PTT at relatively low temperature (41–47 ​°C) by controlling the laser irradiation dosage [292]. After PDT/PTT, owing to the additional immune stimulators released from cancer cells such as immune adjuvants (e.g. granulocyte-macrophage colony-stimulating factor [GM-CSF], ovalbumin [OVA], and oligonucleotides containing cytosine-guanine motifs [CpG]) and proinflammatory cytokines (e.g. IL-12), the tumor-associated antigens are recognized up by the circulating immature dendritic cells (iDCs) to promote the maturation of iDCs. Subsequently, the matured DCs localize in the nearby tumor-draining lymph node and present modified antigen via major histocompatibility complex II (MHCII) to CD4^+^ T helper cells. Through secreting IL-2 cytokine from the activated CD4^+^ T cells, other cytotoxic cells such as cytotoxic T lymphocyte (CTL) could be further activated to destroy the cancer cells. In addition, the memory T cells could also be activated during this process, leading to the tumor killing eventually. However, some immunesuppressive molecules on tumor cells (e.g. programmed death ligands (PDL-1 and PDL-2)), T cells (e.g. CTL-associated protein 4 (CTLA-4)) and programmed cell death protein-1 (PD-1)), can inhibit the CTL-involved anticancer immune response. Therefore, it is usually necessary to administrate the immune-suppressive inhibitors to attenuate the influence of these immunesuppressive factors [293]. The following section attempts to briefly review some recent combinatorial strategies with promising immunotherapeutic effects, particularly focusing on the application of phototheranostic nanomedicine, in the field of phototherapy-synergized cancer immunotherapy.

#### 3.3.1 PTT cancer immunotherapy

**Fig. 20:**
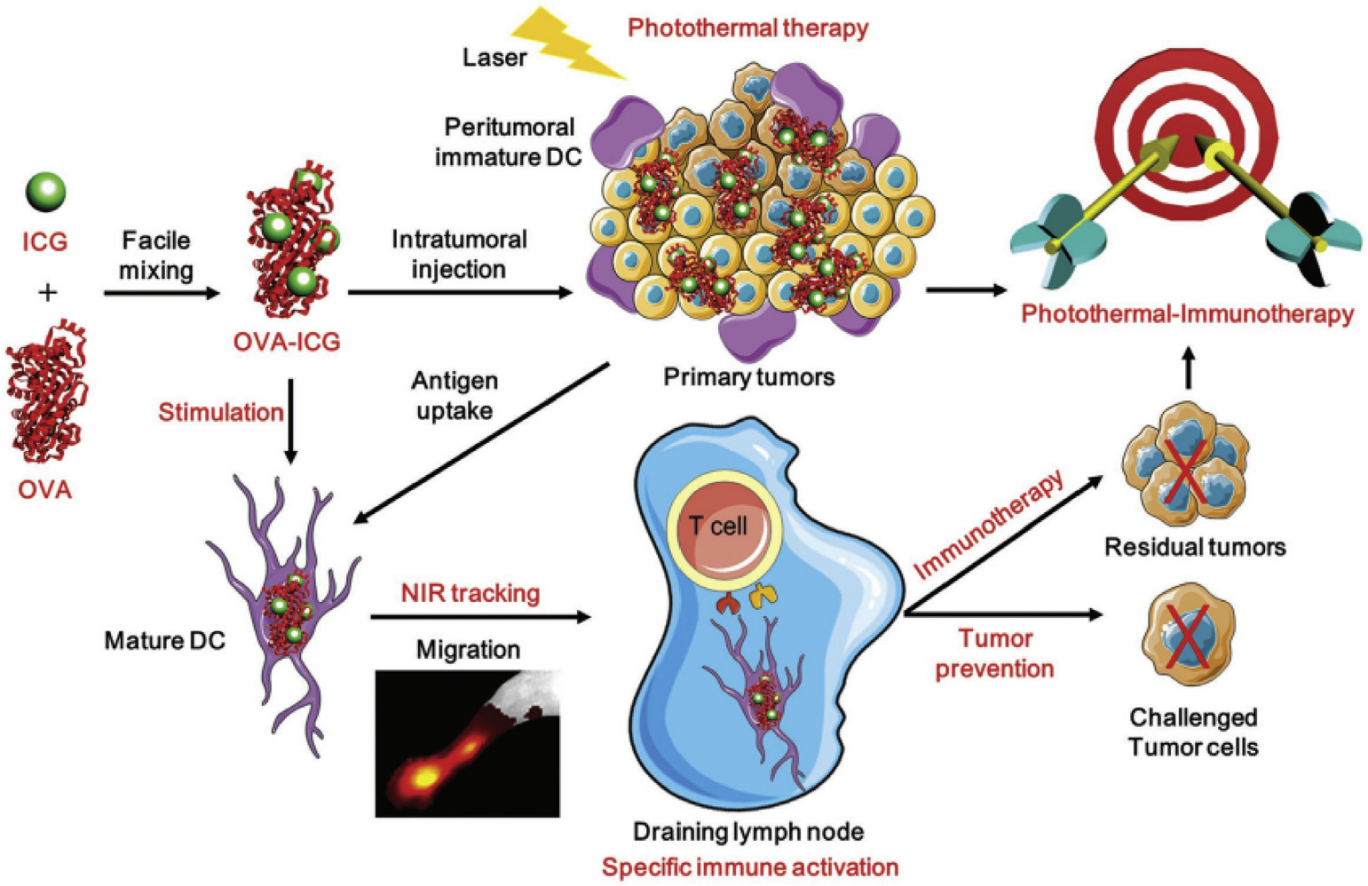
Schematic illustration of the construction of OVA-ICG nanovaccine and its mechanism when applied for PTT-synergized immunoadjuvant cancer therapy [298]. OVA, ovalbumin; PTT, photothermal therapy;ICG, indocyanine green.

In the past few years, various research groups have observed synergism in combining the PTT with other immunotherapeutics. During PTT-mediated cancer immunotherapy, tumor ablation induced by photothermal heating could reduce the difficulty for immune cells to arrive at the TME, which is important for antitumor immune response. It is also worth mentioning that the most suitable temperature range for PTT cancer immunotherapy is from 39 ​°C to 45 ​°C ​becasue that the rapid necrosis of cells caused by higher temperatures could hamper the immunostimulatory process, such as the release of HSPs [294]. Among various strategies, PTT-synergized immunoadjuvant therapy could obtain increased recruitment of lymphocytes, leading to an enhanced cancer immunotherapeutic efficacy [[295], [296], [297]]. For example, IFN-γ not only plays a negative influence on cancer cell proliferation and angiogenesis but also facilitates the MHC I expression in antigen-presenting cells (APCs). Therefore, relevant research has been conducted by Yata et al. [296] ​to increase splenocyte IFN-γ production during PTT, by using a CpG-encoded hexapod-like structured DNA-conjugated GNPs nanogels. Besides inorganic NPs, Pan et al. [298] ​developed a unique approach to encapsulate PTT agents ICG into immune adjuvants OVA, leading to the cargo-free phototheranostic nanomedicine for PTT-synergized immune adjuvant therapy. As shown in Fig. 20, through the facile mixing of ICG and OVA, the OVA-ICG nanovaccines were obtained with high antigen-loading efficiency (80.8%) and ICG loading content (19.2%). In addition, the photothermal conversion efficiency of ICG was not influenced by the formulation of OVA-ICG. Based on their results, the secretions of IL-6 and TNF-á by immature DC 2.4 ​cells were promoted *in vitro*, suggesting the positive immunostimulation. *In vivo* study also revealed that melanoma tumors could be completely suppressed after intratumoral injection of OVA-ICG followed by 808 ​nm laser irradiation, which was not observed for other control treatments.

Some other strategies were developed by incorporating immune checkpoints inhibitors into PTT treatment, also achieved the desired results [[299], [300], [301], [302]]. In a study performed by Chao et al. [303], the PEGlated SWNCTs was prepared and used as the PTT agents to induce the thermal ablation of tumor cells. Upon 808 ​nm NIR laser irradiation, the immunostimulators, including tumor-associated antigens, HSPs, and inflammatory cytokines, were released from tumor cells to induce the maturation of DC cells, finally recruiting tumor-specific CD8^+^ T cells. Moreover, the suppressive activity of T_reg_ cells was efficiently inhibited by introducing the anti–CTLA-4 blockade therapy into the treatment process, leading to the enhancement of the ratios of CD4^+^ and CD8^+^ cells to T_reg_ cells. Notably, CD20^+^ tumor-infiltrating B cells may also have a positive effect on facilitating the presentation of tumor-specific antigens. As expected, the growth of tumors has been inhibited ideally for the mice undergoing PTT-synergized immune checkpoint blockade therapy. Finally, the 4T1 cells lung metastasis experimental model was constructed by researchers. Accordingly, 57% of the mice with combination therapy survived 50 days, which was much higher than the mice which received single surgery (25%). As for another sample, Chen et al. [301] proved that PLGA-loaded ICG and R837 adjuvant could enhance the photothermal immunotherapy efficacy in the presence of CTLA-4 blockade *in vivo*. Tumor growth was inhibited upon laser irradiation and an impressive long-term immune memory was also obtained. A similar strategy could also be applied to PDT-mediated laser immunotherapy. Gao et al. [304] used anti–PD-1 antibodies to block the PD-1 receptors after performing IRDye700-streptavidin-biotin-HK peptide (DSAB-HK)–based PDT. Through preventing the undesired interaction of cancer cells and immune cells, enhanced PDT-mediated laser immunotherapy was easily achieved.

In addition, chimeric antigen receptor (CAR)–redirected T lymphocytes (CAR T cells) therapy as one of promising immunotherapy has attracted much more attention on cancer therapy; however, it exhibited modest therapeutic efficacy in solid tumors. To improve the present situation of CAR T therapy against the solid tumor, Chen et al. [305] used PTT to promote tumor infiltration and antitumor activity of CAR T cells. Their results suggested that mild hyperthermia can not only cause the release of tumor-associated antigens and activate the immune system but also destroy the compact structure of tumor tissue and its extracellular matrix, which would reduce interstitial fluid pressure ​and accelerating the infiltration and accumulation of CAR T cells in the tumor. Thus, the combination of PTT with the adoptive transfer of CAR T cells can potentially improve the therapeutic efficiency of CAR T cells in solid tumors.

#### 3.3.2 PDT cancer immunotherapy

In addition to PTT, PDT could also induce strong antitumor immune response under the similar mechanism as PTT. Some commonly used immune adjuvants (e.g. GM-CSF, OVA, and CpG) were first ​incorporated into the phototheranostic nanosystems to realize the PDT-synergized immunoadjuvant therapy. Besides, some replacements of the aforementioned immune adjuvants with immunogenicity were also reported by different research groups to assist phototherapy to induce the immune response. For instance, a kind of Ca^2+^ binding protein, named calreticulin (CRT), was usually generated in preapoptotic cells. During the cell apoptotic process, CRT within the lumina of the endoplasmic reticulum could be translocated to the cell surface, serving as a phagocytic signal [306,307]. Recently, Wang et al. [308] ​developed an UCNP-based antigen-capturing nanosystem (UCNP/ICG/RB-mal NPs) to augment the antitumor effect of phototherapy. Based on the results, tumor-derived protein antigens, particularly CRT, arising from tumor cells after phototherapy, can be arrested and kept in situ, leading to the improved tumor antigen uptake and presentation by APCs. Synthetic long peptide (SLP) with the immunotherapeutic effect is another kind of novel immune adjuvants, which have been developed as a cancer vaccine for various types of cancer. Kleinovink et al. [309] attempted to combine SLP with bremachlorin-mediated PDT to improve the CD8^+^ T cells percentage and further immunotherapeutic efficacy. The SLP-PDT vaccination remedied over 30% of primary and distant tumors, which was significantly better than the single SLP vaccination (20%) and PDT (0%), demonstrating the acquired systemic immunity.

**Fig. 21:**
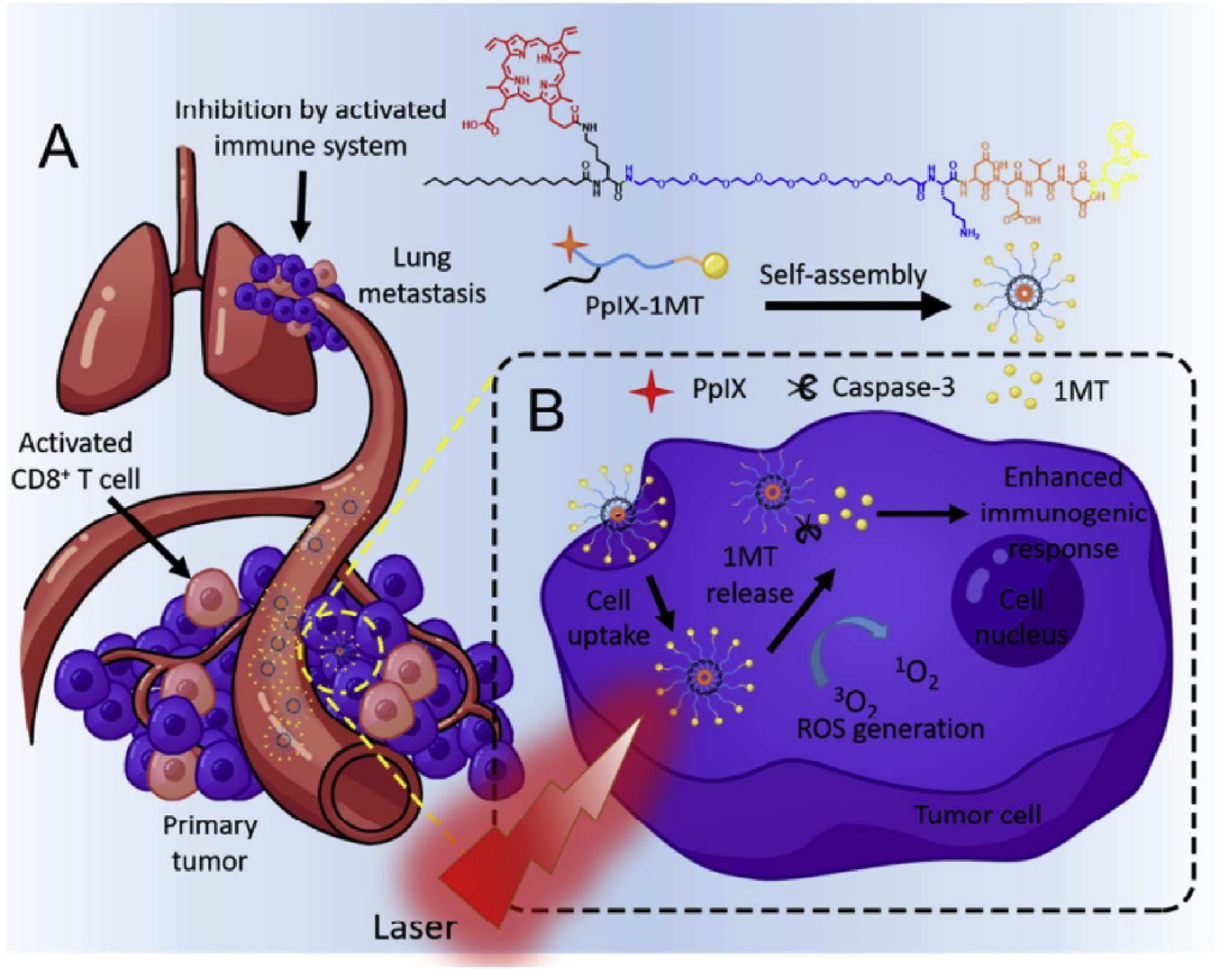
(A) The chemical structure of chimeric peptide PpIX-1MT. (B) Schematic illustration of PpIX-1MT nanoparticles–mediated PDT-synergized checkpoint blockade therapy [317]. PDT, photodynamic therapy.

In addition, there are numerous studies focusing on developing PDT-synergized immune checkpoint blockade therapeutic nanosystems [[310], [311], [312], [313], [314]]. Wang et al. [310] designed an acid-responsive multifunctional micelleplex nanoplatform (named POP micelle), in which PD-L1 was downregulated by siRNA silencing. The adaptive immune response was successfully induced, although facilitating the secretion of proinflammatory cytokines and the recruitment of cytotoxic CD8^+^ T cells. Combing the PD-L1 immune checkpoint blockade therapy with PDT achieved complete tumor eradication, although single PDT or PD-L1 siRNA silencing only inhibited about 73% and 65% of the respect tumor growth, respectively. More recently, indoleamine-2,3 dioxygenase (IDO), an intracellular enzyme overexpressed in the TME of many cancers, was proved to be another promising immune checkpoint modulator [315]. In the presence of IDO, the amino acid tryptophan can be catabolized into kynurenine, leading to the “starvation” of cytotoxic T cells and activation of T_reg_ cells. Accordingly, various IDO inhibitors have been developed, as another strategy for immune checkpoint blockade [316]. Song et al. [317] ​reported the PpIX-1MT NPs self-assembled from the amphiphilic chimeric peptide PpIX-1MT (Fig. 21). As for the chemical structure of PpIX-1MT, an IDO inhibitor, 1-methyltryptophan (1-MT), was conjugated to the C terminal of a caspase-responsive peptide first. Then the N terminal of the peptide was further linked to the PS PpIX through a PEG segment and palmitic acid. As per their results, the 1-MT release behavior from PpIX-1MT NPs in the presence of caspase-3, produced by PDT-induced apoptotic cells, was steady and rapid, up to 83% over 50 ​h. After PDT, the exposure of CRT was enhanced which was detected by flow cytometry, indicating the activation of the immune response during PDT. Meanwhile, the higher ratio of CD8^+^ T cells to CD4^+^ T cells was observed in the serum and spleen. The researchers further proved that the treatment of primary and secondary CT26 tumors in a CT26 metastatic mouse model was successful.

As described previously, although PDT can activate the immune system and be a promising approach to combine other treatments, their therapeutic efficiency was still hardly performed because of poor tumor penetration of PS and other therapeutics. Therefore, Yu et al. [318] designed and prepared an RBC membrane (RBCm)–coated and hyaluronidase-sensitive size-reducible vehicle (mCAuNCs@HA) to realize the combination of PDT and immunotherapy. In this system, when the original vehicle arrived at the tumor tissue, its HA shell would be hydrolyzed and released the inner NPs with the optimal size of 150 ​nm, which were also loaded with PS pheophorbide A (PheoA), ROS-responsive prodrug PXTK and anti-PD-L1 peptide. Thus, with enhanced penetration capacity, this strategy would further enhance antitumor and antimetastasis efficiency through the combination of PDT, immunotherapy, and chemotherapy.

## 4 Summary and perspectives

In this review, we deliver the recent progress about phototheranostic nanomedicine in advanced cancer treatment. PDT was first ​introduced with emphasis on its principle (photophysical mechanism and biological mechanism) and a variety of efficient PSs. Then, PTT and different types of PTT agents are summarized in detail. Phototheranostic nanomedicine has a great potential in cancer therapy and so far abundant photonanoagents have been developed, including polymer-based NPs, liposomes, nanogels, dendrimers, non-biodegradable NPs, and some other novel NPs, which are also described comprehensively in this review with the purpose of providing the reader an overview about the diversity of phototheranostic nanomedicine. Finally, some particular phototherapy-involved biomedical applications in the last decade, such as PA imaging, phototherapy against CSCs and phototherapy-synergized cancer immunotherapy, are briefly discussed. However, although the tremendous amount of promising results are reported in this field in the past decades, there are still many challenges for their further clinical translation into cancer phototherapy.

(1) One of the most important issues for phototherapy is the limited light penetration depth. During PTT, PTT agents with absorption in the BWs, especially in the NIR-II (1000–1400 ​nm), are preferred instead of those absorptions at a shorter wavelength. As for PDT, the visible light used in conventional PDT is also not the desired light source. Although NIR-induced PDT has been reported, such as the use of UCNPs or PDT agents with two-photon effect, great efforts are still required to discover the new generation of PDT agents that can produce ROS efficiently upon being irradiated by the NIR light.
(2) The development of medical equipment that can deliver light into deeper tissues is also required for improving the performance of phototheranostic nanomedicine. It has been proved that the effective penetration depth of NIR light is not deeper than 1 ​cm, even if NIR light is used as the light source. Indeed, for some kinds of cancers, such as skin cancers and oral cancer, light irradiation can be achieved at the tumor sites with the help of optical fibers and the successful therapeutic outcome could be obtained. Unfortunately, the PDT therapeutic efficacy was compromised for some other deeply located cancers. Such phototherapeutic medical equipment may greatly promote the phototherapy to be applied in the clinic, with the efforts of researchers with professions from different fields.
(3) Another considerable challenge in phototherapy of cancer is to deliver the light accurately to the tumors, which may benefit from the well-engineered NPs that render both therapy and imaging functionalities. As aforementioned, PA imaging has great potential in biomedical applications, but the exploitation of more efficient PA imaging agents is essential and still huge challenges remain. Besides, researches on improving PA imaging technology and developing more efficient PA imaging agents are needed to move forward PA imaging to preclinical and clinical applications.
(4) Each phototherapeutic modality has its own advantages and limitations. As for the PDT, the generation of ^1^O_2_ for inducing cell death is largely inhibited in the intrinsic hypoxic tumor environment. Furthermore, this hypoxia level in tumors is usually aggravated during the process of PDT because of the consumption of oxygen. To improve the therapeutic efficacy by PDT, it has to enhance the light exposure time and laser power, which may damage normal tissues and impede the further clinical application of PDT. As for PTT, the induction of molecular chaperones, known as HSPs, has frequently compromised the PTT effect *in vitro* and *in vivo*, particularly when non-lethal thermal dose used during the therapy. Thus, the combined phototherapy with other treatments (e.g. chemotherapy) may be a promising strategy for ideal tumor therapy, which could be achieved by the development of multifunctional nanomedicine.
(5) As for the phototherapy-synergized cancer immunotherapy, it is necessary to discover more efficient immune adjuvants and checkpoints to render better modulate of immunesuppression during the therapy. For instance, one potential immune checkpoint is an integrin-related receptor overexpressed on the surface of various tumor cells, CD47. Through blocking the CD47 receptor by anti-CD47 antibody, the interaction between cancer cells with signal regulatory protein (SIRP)-alpha on APCs could be interrupted, further activating the antitumor activity [319]. Besides, involving more immunotherapeutic modalities into phototherapy could provide more options for the phototherapy-synergized cancer immunotherapy. CAR-redirected T lymphocytes (CAR T cells) has shown limited therapeutic outcome in solid tumors because of the desmoplastic structure of the tumors and the immunosuppressive TME. Chen et al. [305] ​have combined PTT-mediated mild hyperthermia with the adoptive transfer of CAR T cells for efficient cancer treatment. The results demonstrated that PTT facilitated the collection of CAR T cells within solid tumors to achieve the desired therapeutic efficacy, through reducing high interstitial ﬂuid pressure of tumor, increasing blood perfusion, releasing antigens, and promoting the recruitment of cytotoxic CD8^+^ T cells.
(6) The use of nanomedicine for cancer diagnosis and treatment has attracted considerable attention over the past two decades. However, it is a fact that there are still various biological barriers that are faced by these multimodal systems in the delivery to the target site, slowing their translation into the clinical environment. For example, the RES ​plays crucial roles in NP clearance. Aside from the PEGylation of NPs to hinder opsonization, coating them with the cell membrane derived from erythrocytes or leukocytes displayed promising advantages on minimizing non-specific clearance of NPs by RES. In this strategy, a highly efficient extraction of cell membranes and the universal application of this technique on different types of NPs are still much inadequate to hinder successful clinical translation. In addition to the RES, renal ultrafiltration also influences the pharmacokinetics of NPs. Experimental results show that the size, shape, and surface charge of NPs are closely relevant to renal excretion, guiding researchers to develop a large number of nanosystems with ideal metabolic properties. However, it is more complicated for patients with renal insufficiencies (e.g. chronic kidney diseases) or patients administrated of nephrotoxic chemotherapeutics (e.g. cisplatin), highlighting the necessity for a personalized evaluation of renal excretion profile of different nanosystems. The BBB, protecting the central nervous system (CNS), is a non-negligible obstacle for drug delivery for CNS-related carcinoma. Unlike pharmaceutical compounds, NPs can cross BBB via receptor-mediated endocytosis. Although much attention has been paid to optimize the BBB penetration of NPs, few have assessed how these nanosystems would be metabolized, decomposed, and removed from CNS.

Aside from the aforementioned challenges, there are also plenty of mechanism-associated issues that remain to be addressed along with the development of phototheranostic nanomedicine. Even though, it is believed that phototherapy by using functional nanoplatforms would play more important roles in the cancer treatment in the future because of their particular advantages [107,108]. What is particularly noteworthy is the fact that the phototherapy has also been used to treat other types of pathologies, such as Alzheimer's disease [320], transient acantholytic dermatosis (Grover's disease) [321], psoriasis [322], vitiligo [323], and so on. Therefore, the application range of the phototheranostic nanomedicine discussed in this review would be continuously extended in the near future.

## Author contributions

D. G., X. G., and X. Z. contributed equally to this work. D. G., X. G., and X. Z. wrote this manuscript; D. G., X. G., S. C., Y. W., T. C., G. H., and X. Z. collected and classified the references; the review was performed based on the direction of Z. Y., X. Z., Z. T., and Y. G.

## Declaration of competing interest

The authors declare that they have no known competing financial interests or personal relationships that could have appeared to influence the work reported in this article.
